# A comprehensive water buffalo pangenome reveals extensive structural variation linked to population-specific signatures of selection

**DOI:** 10.1093/gigascience/giaf099

**Published:** 2025-08-30

**Authors:** Fazeela Arshad, Siddharth Jayaraman, Andrea Talenti, Rachel Owen, Muhammad Mohsin, Shahid Mansoor, Muhammad Asif, James Prendergast

**Affiliations:** The Roslin Institute, University of Edinburgh, Easter Bush, Midlothian EH25 9RG, UK; Agricultural Biotechnology Division, National Institute for Biotechnology and Genetic Engineering College (NIBGE-C), Faisalabad, 38000, Pakistan; Pakistan Institute of Engineering and Applied Sciences (PIEAS), Nilore, Islamabad, 45650, Pakistan; The Roslin Institute, University of Edinburgh, Easter Bush, Midlothian EH25 9RG, UK; The Roslin Institute, University of Edinburgh, Easter Bush, Midlothian EH25 9RG, UK; The Roslin Institute, University of Edinburgh, Easter Bush, Midlothian EH25 9RG, UK; Agricultural Biotechnology Division, National Institute for Biotechnology and Genetic Engineering College (NIBGE-C), Faisalabad, 38000, Pakistan; Pakistan Institute of Engineering and Applied Sciences (PIEAS), Nilore, Islamabad, 45650, Pakistan; Jamil ur Rehman Centre for Genome Research, International Centre for Chemical and Biological Sciences, University of Karachi, Karachi 75270, Pakistan; Agricultural Biotechnology Division, National Institute for Biotechnology and Genetic Engineering College (NIBGE-C), Faisalabad, 38000, Pakistan; Pakistan Institute of Engineering and Applied Sciences (PIEAS), Nilore, Islamabad, 45650, Pakistan; The Roslin Institute, University of Edinburgh, Easter Bush, Midlothian EH25 9RG, UK

**Keywords:** water buffalo, genome assembly, pangenome, structural variation, Pakistani river buffalo, Nili-Ravi, Azikheli

## Abstract

**Background:**

Water buffalo is a cornerstone livestock species in many low- and middle-income countries, yet major gaps persist in its genomic characterization—complicated by the divergent karyotypes of its two subspecies (swamp and river). Such genomic complexity makes water buffalo a particularly good candidate for the use of graph genomics, which can capture variation missed by linear reference approaches. However, the utility of this approach to improve water buffalo has been largely unexplored.

**Results:**

We present a comprehensive pangenome that integrates 4 newly generated, highly contiguous assemblies of Pakistani river buffalo with 8 publicly available assemblies from both subspecies. This doubles the number of accessible high-quality river buffalo genomes and provides the most contiguous assemblies for the subspecies to date. Using the pangenome to assay variation across 711 global samples, we uncovered extensive genomic diversity, including thousands of large structural variants absent from the reference genome, spanning over 140 Mb of additional sequence. We demonstrate the utility of these data by identifying putative functional indels and structural variants linked to selective sweeps in key genes involved in productivity and immune response across 26 populations.

**Conclusions:**

This study represents one of the first successful applications of graph genomics in water buffalo and offers valuable insights into how integrating assemblies can transform analyses of water buffalo and other species with complex evolutionary histories. We anticipate that these assemblies, as well as the pangenome and putative functional structural variants we have released, will accelerate efforts to unlock water buffalo’s genetic potential, improving productivity and resilience in this economically important species.

## Background

Water buffalo (*Bubalus bubalis*; NCBI:txid89462) are central to the livelihoods of millions of people worldwide, especially in low- and middle-income countries (LMICs). Among these, Pakistan, India, China, and several Southeast Asian nations rely heavily on water buffalo for milk, meat, and draft power. In Pakistan alone, buffalo contribute around 60% of the total milk production [1]—underscoring their critical role in national food security and the incomes of smallholder farmers. Beyond its contributions to nutrition and the agricultural economy, water buffalo hold cultural importance in many regions, where they represent a valuable, multipurpose asset that can thrive in diverse ecological conditions.

Despite its socioeconomic significance, water buffalo have historically received less systematic genomic research compared to its bovine relative, the domestic cow (*Bos taurus/Bos indicus*). This is partially due to the lower use of water buffalo in high-income countries, resulting in more limited investment into genomic research and breeding programs. Consequently, key questions remain unanswered, including how different alleles and structural variants (SVs) influence key traits like milk yield, carcass quality, growth rate, and disease resistance. Addressing this could help unlock the genetic potential of water buffalo and dramatically enhance production efficiency, particularly benefiting smallholder farming communities in LMICs.

A major factor complicating water buffalo genomic analyses is the species’ complex evolutionary history. Present-day water buffalo descend from wild *Bubalus arnee* [2]. Following 2 distinct domestication events of separate buffalo populations, 2 primary subspecies emerged: river buffalo (*Bubalus bubalis bubalis*, 2n karyotype = 50), widely used for high-yield milk production, and swamp buffalo (*Bubalus bubalis carabanensis*, 2n = 48), primarily used for draft and meat [3]. Although they exhibit distinct karyotypes, both subspecies can interbreed and produce fertile offspring [4]. Yet this divergent chromosome number, estimated to have originated around 3 million years ago, complicates analyses of genomic variation [5]. For example, the water buffalo genome that to date has been most commonly used as a reference is derived exclusively from a river buffalo [6], which can lead to inaccuracies when aligning swamp buffalo sequences. Swamp populations—that predominate in mainland Southeast Asia—show higher levels of divergence and harbor structural variants not captured by a river-centric reference [5], resulting in biases in variant calling and downstream analyses. Although swamp buffalo assemblies are now available (e.g., [5]), no single linear reference can capture the diversity across the species.

Capturing these large-scale genomic differences is crucial for understanding phenotype variation, such as in relation to milk production, meat quality, and disease resistance. Structural variants (such as insertions, deletions, and inversions) encompass more nucleotides than single-nucleotide polymorphisms (SNPs) [7], consequently potentially having larger impacts on heritable phenotypes. Studies in other livestock species have highlighted the importance of identifying such variants [8]. However, for water buffalo, the scarcity of high-quality genome assemblies, especially from regions with richly diverse indigenous breeds, has slowed progress. Although 8 comparatively high-quality long-read–based assemblies in terms of contiguity (contig N50 > 1 Mb) are publicly available—[5, 6, 9–12] split evenly between the swamp and river types—none were generated from Pakistani buffalo, despite the importance of local breeds like Nili-Ravi and Azikheli to milk production in South Asia [13, 14].

Over the past decade, several studies have investigated possible selective sweeps in water buffalo breeds by analyzing SNPs derived from genotyping arrays or short-read whole-genome sequencing data aligned to a single river buffalo reference [15–17]. These efforts have identified loci putatively linked to economically and biologically important traits, including lactation, fertility, growth, and immune response. For example, Dutta et al. [15] used whole-genome sequencing and population genomic analyses to detect selective signatures in Indian river buffalo, while Sun et al. [17] and Si et al. [16] similarly reported candidate genomic regions under positive selection in both swamp and river lineages. Such studies highlight how domestication pressures and local adaptations have shaped the water buffalo genome in different geographical contexts. Despite these advances, these investigations of selective sweeps in water buffalo have focused on short variation. Whether any larger variants may underlie selective sweeps in the species largely remains unexplored.

Graph genomics offers a powerful alternative to traditional linear reference-based analyses. By integrating multiple assemblies into a single “pangenome,” graph methods can more accurately represent breed- or subspecies-specific haplotypes, including large structural variants [18, 19]. This avoids biases inherent in mapping reads to a single reference, particularly when working with genetically diverse or structurally distinct populations. Graph-based approaches have reduced false-negative rates in larger variant calling for other livestock [20–22], revealing alleles that were previously hidden when aligned to a single linear reference. Yet, such methods have largely not been applied to water buffalo, leaving potentially important functional variation in the species uncharted.

Here, we address this gap by generating new, highly contiguous assemblies for Pakistani river buffalo, significantly expanding existing genomic resources. We construct the first comprehensive pangenome that includes these new assemblies alongside publicly available river and swamp buffalo genomes. By genotyping global water buffalo samples against this pangenome, we generate the largest combined water buffalo reference set of structural and single-nucleotide variation to date and identify previously unidentified structural variants potentially underlying natural and artificial selective sweeps. Collectively, this work has the potential to inform a diverse range of studies, including breeding programs, conservation strategies, and future genomic analyses, ultimately improving water buffalo productivity and resilience worldwide.

## Methodology

### Sample collection and DNA extraction

The animal handling and sample collection protocol was reviewed and approved by the Research Ethics Committee of the National Institute for Biotechnology and Genetic Engineering (NIBGE), Faisalabad, Pakistan, on 29 May 2024. One female Nili-Ravi buffalo from the Punjab province of Pakistan and 1 Azikheli female buffalo from Swat, a district of the province of Khyber Pakhtunkhwa, Pakistan, was selected for genome sequencing (Supplementary Figs. S1, S2). Blood sample collection was conducted under the supervision of trained animal care specialists to minimize stress and ensure the welfare of the animals. Fresh blood was collected from the jugular vein of animals in EDTA-coated tubes and kept cool on ice gel packs for transportation to the laboratory at NIBGE. Genomic DNA was isolated using the Thermo Scientific GeneJET Whole Blood Genomic DNA Purification Mini Kit following the manufacturer’s protocol. Prior to extraction, blood was preprocessed by freshly prepared digestion solution-A (1 M MgCl_2_,1 M Tris-HCl [pH 7.5], 2 M sucrose, and Triton X-100) with the aim of increasing the yield. In total, 750 µL of thoroughly homogenized blood was transferred to a sterile 1.5-mL microcentrifuge tube, and an equal volume of “solution-A” was added. The mixture was vortexed and incubated at room temperature for 10 minutes. It was then centrifuged at 11,000 rpm for 45 seconds. After centrifugation, the supernatant was discarded, and the pellet was resuspended in 400 µL solution-A. This process of incubation at room temperature for 10 minutes, followed by centrifugation at the same speed for 45 seconds, was repeated until the supernatant became clear. Following the final removal of the supernatant, the pellet was dissolved in 190 µL phosphate-buffered saline (PBS) to achieve a final volume of 200 µL. This 200-µL white blood cell (WBC) suspension in PBS was subsequently used for DNA extraction following the kit protocol, instead of using whole blood.

### Sequencing and genome assembly

Extracted DNA was shipped to the Edinburgh Genomics sequencing facility at the University of Edinburgh in the United Kingdom, and PacBio HiFi sequencing data were generated using PacBio Revio SMRTbell library preparation.

The resulting HiFi data with a quality of Q ≥ 20 were checked with FASTQCv0.12.1 (RRID:SCR_014583) [23]. A total of 98.2 Gbp of sequence data, with a median read length of 15.8 kb, was generated for the Azikheli buffalo (AZ0004), representing a mean depth coverage of ∼34× under an assumed genome size of 2.9 Gbp. Similarly, 102.5 Gbp of sequence data, with a median read length of 17.1 kb, was generated for the Nili-Ravi buffalo (NR0003), achieving a mean depth coverage of ∼35×. The long HiFi fastq reads were *de novo* assembled using HiFiasm v0.24.0-r702 (RRID:SCR_021069) [24] with the -z20 option added to trim 20 bp from both ends of the reads, to produce dual contig-level assemblies (i.e., 2 assemblies per animal). BUSCO (RRID:SCR_015008) [25] completeness for each assembly was assessed using the artiodactyla_odb12 database with BUSCO v5.8.3 and AUGUSTUS v3.5.0 (RRID:SCR_008417) [26]. nf-LO v1.8.6 [27] was used to lift gene annotations from the Mediterranean reference assembly to each novel assembly using minimap2 v2.28 (RRID:SCR_018550) [28] as the aligner and with the –distance “near” parameter. Coding sequence and protein fasta were then generated using gffread v0.12.7 (RRID:SCR_018965) [29].

### Construction of pangenome graph

To make a water buffalo pangenome incorporating both subspecies, 8 publicly available water buffalo genome assemblies with the best assembly statistics were obtained. The river buffalo assemblies were UOA_WB_1 [6], NDDB_DH_1, NDDB_SH_1 [10], and CUSA_RVB [11]. The swamp buffalo assemblies were BBCv1.0 [9], PCC_UOA_SB_1v2 [5], CUSA_SWP [11], and Wang_2023 [12]. These genomes were accessed and downloaded from the National Center for Biotechnology Information (NCBI), China National Gene Bank (CNGB), and National Genomics Data Center (NGDC) databases. Further information relevant to the accession numbers can be found in the Data Availability section.

To eliminate potential biases arising from the differences in tools and versions, and to ensure fair comparison between our novel genome assemblies and publicly available genome assemblies, we recalculated the assembly statistics for all genomes using gfastats v1.3.6 (RRID:SCR_026368) [30].

The PanGenie Snakemake pipeline [31] was used for the construction of the graph genome from our 4 newly generated haplotype-resolved assemblies NIBGE_UOEAWB_hap1 (Azikheli 1), NIBGE_UOEAWB_hap2 (Azikheli 2), NIBGE_UOENRWB_hap1 (Nili-Ravi 1), and NIBGE_UOENRWB_hap2 (Nili-Ravi 2) and the 7 publicly accessed genomes NDDB_DH_1 (Indian Murrah 1), NDDB_SH_1 (Indian Murrah 2), CUSA_RVB (Chinese Murrah), CUSA_SWP (Zhuang female), BBCv1.0 (Chinese swamp), PCC_UOA_SB_1v2 (Philippines swamp), and Wang_2023 (Zhuang male). This pipeline involves aligning contigs from each assembly to the chosen reference genome using minimap2 (RRID:SCR_018550) [28], then using minimap2’s paftools to call variants for each assembly in callable regions, defined as portions of the reference where just 1 contig aligns. These single-assembly variant call sets are then merged to produce a pangenome graph represented as a multiallelic, multisample variant call file (VCF) file. UOA_WB_1 [6] was chosen as the reference genome for this analysis given its comparative widespread use in previous studies and it being listed as the species reference on Ensembl. Since the 2 buffalo subspecies have different chromosome numbers (river 2n = 50 and swamp 2n = 48) originating from a fusion of river buffalo chromosomes 4 and 9 [32], chromosome 1 of the swamp assemblies was split at the point of the fusion prior to running the PanGenie Snakemake pipeline. The fusion point was identified using pairwise alignment format (PAF) files, which provided start and end coordinates of the aligned regions. The midpoint of the gap between these alignment regions was calculated to determine the fusion point. The same approach was applied to all swamp assemblies, and the Snakemake workflow was then executed with the updated data.

The resultant multiallelic, multisample VCF was normalized using the bcftools v1.20 (RRID:SCR_005227) [33] norm function. To quantify the variation specific to each combination of assemblies, a support vector (SUPP_VEC) field was introduced into the VCF file to record the presence (“1”) or absence (“0”) of the alternative allele for each variant in every sample. Bcftools v1.13 (RRID:SCR_005227) [33] was used to query support vector values for variants with lengths over 50 bp, ensuring structural variant information was captured. The resulting data were plotted using the UpSetR package [34] to illustrate the size distribution of structural variants across samples. Following this, to calculate the extra sequence lengths in the buffalo pangenome, the normalized graph filtered VCF file was processed to include length annotations by utilizing the vcflength tool within the vcflib v1.0.12 (RRID:SCR_001231) [35] environment. The insertion lengths for heterozygous or alternative homozygous variants were extracted for each sample of interest by querying the relevant data with bcftools v1.13 (RRID:SCR_005227). The total lengths contributed by different assemblies were calculated with the help of a Python script.

For the phylogenetic relationship of the assemblies, we generated a mash distance (distance matrix)–based phylogenetic tree from the fasta sequences of studied water buffalo assemblies with the cattle reference *B. taurus* ARS UCD 2.0 [36] genome added as an outgroup, using mashtree v1.4.6 [37] with the options –reps 100 and –min-depth 0 to increase bootstraps and ignore low abundance *k*-mers. Tree visualization was done using figtreev1.4.4 (RRID:SCR_008515) [38].

### Whole-genome sequence data across buffalo populations

The structural variants identified in our pangenome graph were genotyped across a larger cohort of 711 whole-genome sequences from diverse global buffalo populations. Whole-genome sequences were accessed from projects PRJNA633724 [39], PRJEB39591 [15], PRJNA547460 [17], PRJCA001294 [11], PRJNA350833 [40], PRJNA1135737, PRJNA1057008, and PRJNA633919 [16]. These data were downloaded from the NCBI, NGDC, and European Nucleotide Archive (ENA) databases. The 711 water buffaloes from 16 countries included 337 domesticated river buffalo and 374 domesticated swamp buffalo (Supplementary Table S1). Both subspecies were further classified into subgroups based on their geographical distribution. The river buffalo were divided into 6 geographical groups: South Asia (SA), Italy (ITA), West Asia (WA), Egypt (EGY), Nepal (SA-NP), and South Bangladesh (BGD-S). Similarly, the swamp buffalo populations were categorized into Central China (CHN-CE), Southwestern China (CHN-SW), Northeast Bangladesh (BGD-NE), Southeast China (CHN-SE), Southeast Asia (SEA), and Indonesia (IND).

### PanGenie genotyping and data filtering

To mitigate against poor-quality genotyping, we included only samples with Illumina, paired-end mean read coverage greater than 10× resulting in the abovementioned sample size of 711 individuals. Fastq reads from the NCBI, ENA, and NGDC databases were downloaded using enaBrowserTools v1.7.1 [41]. The VCF for each unique biosample were generated using PanGenie v3.0.0 [31]. PanGenie re-genotyped variants provided in the input graph VCF file derived from the 12 assemblies and referenced to the UOA_WB_1 reference assembly, ensuring that the output VCF contains the same variant records as the input, but with genotypes assigned for the sample on which PanGenie is run. The quality of each genotyped VCF was checked by calculating statistical parameters using RTG Tools v3.12.1 [42]. Resultant genotyped files for each biosample were merged using the “–region option” in bcftools v1.13 (RRID:SCR_005227). For downstream analyses, variants with >20% missing genotypes and minor allele frequency <5% were excluded using bcftools options “view -i F_MISSING<0.2” and “view -i 'MAF>0.05.”

### Relatedness

To assess the population structure of the study cohort, the 711 samples were subjected to kinship analysis to exclude related individuals, monozygotic duplicates, and sequencing artifacts, thereby ensuring an accurate representation of the genetic pool. This was accomplished by measuring kinship coefficients using King v2.1.2 (RRID:SCR_009251) [43] with options –kinship and –degree 3. To identify and filter related individuals, kin pairs closer than third-degree (KING kinship >0.0625) were pruned. Individuals with kinship values above this threshold were grouped into connected components, representing clusters of related individuals. Within these clusters, individuals with higher mean read depth coverage were prioritized and categorized in the “keep” group, while those with comparatively lower coverage were placed in the “remove” group and excluded. This process generated a list of 403 unrelated individuals. Since after the exclusion of certain samples, the minor allele frequency (MAF) can change, unrelated samples were refiltered for MAF >0.05. Before performing principal component analysis (PCA) and admixture analyses, linkage disequilibrium (LD) -based pruning was performed using plink 1.9 (RRID:SCR_001757) [44] with the –indep pairwise 50 10 0.2 options with an additional option of –mind 0.20 to exclude samples with a missing genotype rate >0.20. Then we estimated eigenval and eigenvec values using plink 1.9’s –pca option. Admixture v1.3.0 (RRID:SCR_001263) [45] was run with the number of assumed ancestral populations (K) ranging from 2 to 9, with K = 6 identified as the best model based on cross-validation (CV) values (Supplementary Fig. S3).

### Genotype concordance

To compare variant calls between PanGenie [31] and GATK (RRID:SCR_001876) [46], we used the 81 samples from a previous study for which GATK calls were also available [15]. Note that 2 samples were technical replicates in this dataset, meaning it contained 79 distinct animals. Variants were hard filtered as previously described. Importantly, this did not involve the use of GATK’s VQSR, which may bias the results due to its dependence on existing sets of known variants. Genotypes were compared using RTG tools v 3.12.1 [42] run in its default “weighted” mode, having first applied a further GQ filter of ≥20 to both VCF files. Because vcfeval’s weighted scheme prevents double-counting when allele representations differ, the percentages convey true biological concordance rather than artifacts of VCF formatting. To determine which variants fell within repetitive regions, repeat masker annotation was downloaded from the University of California, Santa Cruz website. Hardy–Weinberg equilibrium values and allele frequencies were calculated using bcftools v1.19 (RRID:SCR_005227). Allele frequencies of PanGenie- and GATK-specific variants were calculated only at variants where at least 50 samples had a genotype call in the respective callset, and this analysis was restricted to 3 representative chromosomes of differing sizes (2, 12, and 22).

### Selective sweep analyses

For the selective sweep analyses, the 403 samples filtered for relatedness were grouped based on their breed labels, sampling location, and position on the PC1 versus PC2 PCA. This resulted in 26 groups with at least 6 samples, encompassing a total of 282 samples, that were taken forward for analysis (Supplementary Table S2). Including close kin could otherwise inflate the long-range haplotype homozygosity statistics and create false-positive sweep signals. Following filtering out genotypes with a genotype quality less than 25 using bcftools [33], the dataset was further restricted to 31,504,264 biallelic variants with genotypes present for at least 75% of samples, filtering out 1,316,911 multiallelic variants. The resulting VCF was then phased using Beagle v5.2 [47] (RRID:SCR_001789), and the integrated haplotype score (iHS) and number of segregating sites by length (nSL) statistics were calculated for each variant and group using HapBin v1.3.0 [48] and Selscan v2.0.3 [49], respectively. Putative sites of selective sweeps were called as previously [15]. For both the iHS and nSL results, scores were first averaged across 100 variant windows, and peaks were then called where the absolute of these mean values rose above 1.5 and fell back below 0.5. Genes overlapping these peaks were then identified using a custom R script and the NCBI gene annotations for water buffalo (Bubalus_bubalis-GCA_003,121,395.1–2020_06-genes.gff3).

The variant allele frequency differences for 26 selected population groups were calculated by first normalizing the variants using  bcftools v1.20 (RRID:SCR_005227) [33]–norm function, annotating them with SnpEff v5.2f (RRID:SCR_005191) [50] using the genomic annotation file GCF_003,121,395.1_ASM312139v1_genomic.gff and the reference genome GCA_003,121,395.1_UOA_WB_1_genomic.fna from NCBI, and adding fill-tags using bcftools v1.20 (RRID:SCR_005227) with the focus on high- and moderate-impact variants. Selection regions were defined by merging peak coordinates into unique BED files. Within these regions, alternate allele frequencies (aAFs) were queried for each population using bcftools v1.20 (RRID:SCR_005227), highlighting key high- and moderate-impact variants associated with population-specific genetic adaptations.

## Results

### Novel assemblies for Pakistani breeds

**Figure 1:**
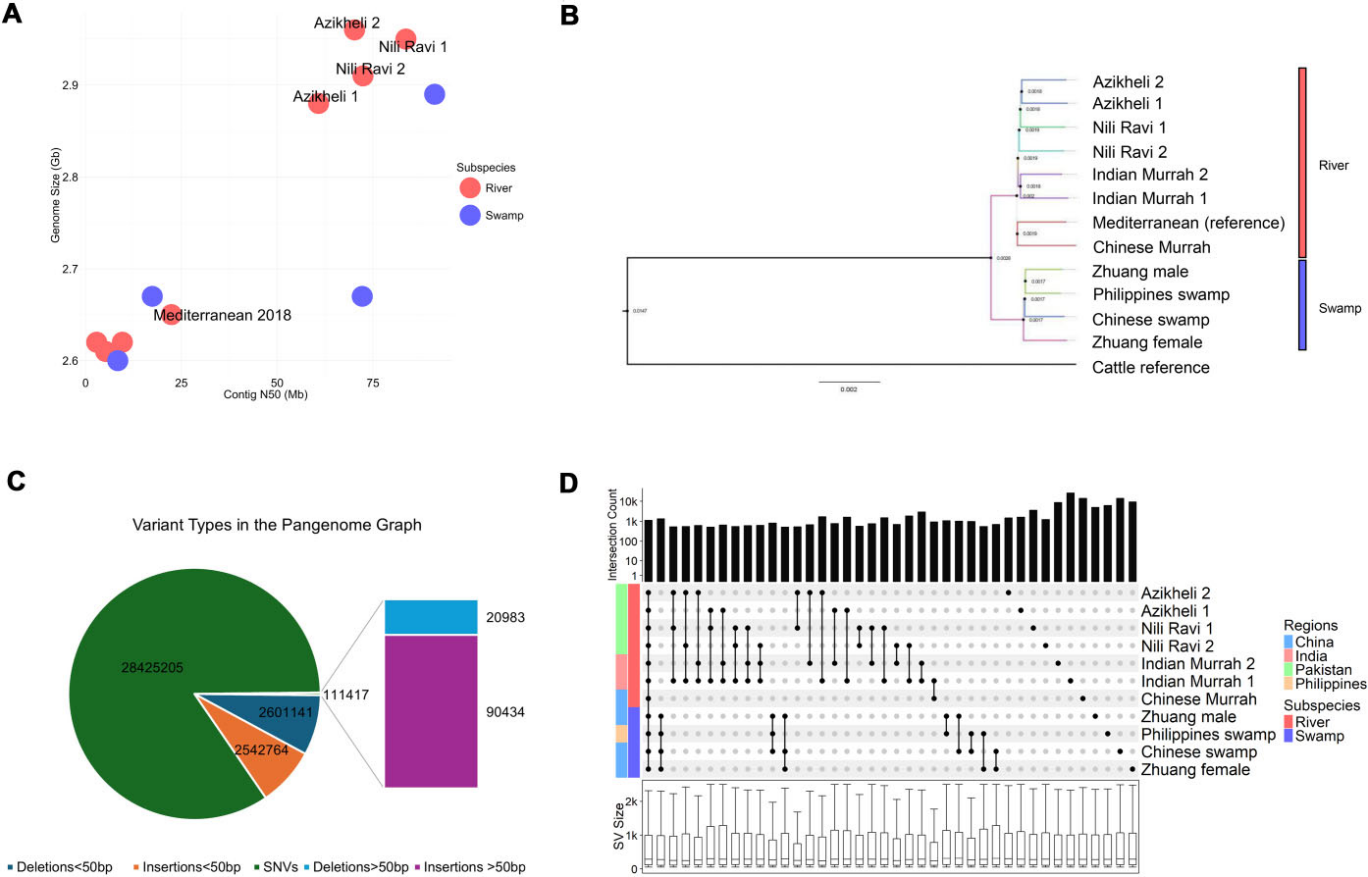
(A) Scatterplot of publicly available and newly generated genome assemblies, illustrating contig N50 on the x-axis and estimated genome size on the y-axis. Red bubbles represent river buffalo genomes, while blue bubbles denote swamp buffalo genome assemblies. The newly generated assemblies are highlighted as the top 4 red-labeled bubbles, surpassing the contiguity of the Mediterranean river genome (also labeled). (B) Phylogenetic tree showing the evolutionary relationships among the 12 water buffalo genome assemblies used in this study, including the newly generated haplotype resolved assemblies at the top and the cattle reference assembly (at the bottom) added as an outgroup. The tree was constructed based on the mash distances (distance matrix) method with 100 bootstrap replicates. Bootstrap values are displayed at the nodes. (C) Proportion of variants in each class in the pangenome graph. The graph includes insertions and deletions (indels) <50 bp and structural variants (SVs) ≥50 bp. (D) Upset plot of sets of SVs found across different assemblies. Each column represents a set of SVs with the points indicating in which assemblies the SVs were found. The bar graph along the top displays the number of SVs in the corresponding set. Only the 40 sets with the most SVs are shown.

To address the current lack of high-quality assemblies for Pakistani water buffalo breeds, we first generated PacBio HiFi reads for 1 female Nili-Ravi buffalo (ID:NR0003) and 1 female Azikheli buffalo (ID:AZ0004), 2 common breeds used in the country [13]. The Nili-Ravi, in particular, corresponds to 38% of the national buffalo population [13]. In total, 102.5 Gbp (∼35× coverage) and 98.2 Gbp (∼34×) of data, respectively, were generated. Using the Hifiasm (v0.24.0-r702) assembler, we produced a pair of dual assemblies for each animal with contig N50s ranging from 61 to 84 Mb and BUSCO [25] completeness scores of 98%. Comparison of the statistics of these novel assemblies to the best currently publicly available genomes confirms that they are among the most contiguous water buffalo genomes generated to date (Table 1, Fig. 1A) and the most complete river buffalo genomes, substantially exceeding UOA_WB_1 in both total size and contiguity. This increase in genome size is consistent with that seen in other species for assemblies generated with the latest sequencing technologies [51], and this and the high BUSCO scores are potentially consistent with repetitive regions being better resolved. Phylogenetic analysis of these novel and publicly available assemblies confirms the split between swamp and river buffalo, with the new Pakistani assemblies clustering as a group and most closely to the existing Indian river buffalo assemblies (Fig. 1B), matching their geographic proximity and in broad agreement with previous studies of short- read sequencing data [16].

**Table 1:** Genome assembly metrics for publicly available and newly generated haplotype resolved genome assemblies. All values were calculated using gfastats v1.3.6 [30] to ensure consistency. Originally reported metrics and assembly methods are compiled in Table 1 of [5] and found to be comparable.

Assembly name	Subspecies	Label	Genome size (Gb)	Contigs	Contig N50 (Mb)	Contig L50	GC content %	Reference
NIBGE_UOEAWB_hap1	River	Azikheli 1	2.88	852	60.8	17	42.84	Current study
NIBGE_UOEAWB_hap2	River	Azikheli 2	2.96	582	70.2	16	43.22	Current study
NIBGE_UOENRWB_hap1	River	Nili-Ravi 1	2.95	510	83.6	14	43.18	Current study
NIBGE_UOENRWB_hap2	River	Nili-Ravi 2	2.91	674	72.4	16	43.01	Current study
UOA_WB_1 (reference)	River	Mediterranean 2018	2.65	918	22.4	36	41.74	[6]
BBCv1.0	Swamp	Chinese swamp	2.67	8,520	17.4	367	41.78	[9]
NADDB_DH_1	River	Indian Murrah 1	2.61	14,366	5.22	1707	41.75	[10]
NDDB_SH_1	River	Indian Murrah 2	2.62	5,265	9.59	636	41.85	[10]
PCC_UOA_SB_1v2	Swamp	Philippines swamp	2.89	297	91.1	29	42.66	[5]
CUSA_SWP	Swamp	Zhuang female	2.60	9,428	8.39	187	41.83	[11]
CUSA_RVB	River	Chinese Murrah	2.62	11,874	2.88	553	41.9	[11]
Wang_2023	Swamp	Zhuang male	2.67	346	72.2	27	41.8	[12]

### Identification of structural variants based on pangenome graph

In order to create the water buffalo pangenome graph and identify SVs, these 4 novel and 7 public water buffalo assemblies (Table 1) were aligned to our chosen reference genome (UOA_WB_1) using PanGenie’s minimap2 pipeline. The resultant pangenome graph contained a total of 32,821,198 variants, of which 2,542,764 and 2,601,141 were insertions and deletions less than 50 bp (indels) long, and 28,425,205 were single-nucleotide variants (SNVs/SNPs). There were a further 111,352 SVs, including 90,434 insertions and 20,983 deletions across the 24 autosomal chromosomes (Fig. 1C). As shown in Fig. 1D, the majority of SVs were found to be unique to individual assemblies. This observation is consistent with previous studies of cattle [52]. In total 1,347 SVs, spanning a total sum of 0.31 Mb, were found specifically across all of the swamp genome assemblies, suggesting these SVs likely represent genomic segments specific to and fixed across this subspecies relative to river buffalo.

Emphasizing the divergence of the 2 subspecies, among the top 40 sets of SVs shown in Fig. 1D, only 1 set involved SVs shared across swamp and river assemblies—the set where the SVs were found in all of the nonreference assemblies—suggesting the variant is private to the chosen reference assembly. Consequently, there is comparatively little SV sharing across subspecies.

In total, this buffalo pangenome graph contained an extra 147,865,364 bases in paths not present in the reference genome (Supplementary Table S3). The 7 river assemblies exclusively contributed 73,672,251 bases, slightly higher than the exclusive 70,756,474 bases contributed by the fewer 4 swamp assemblies. Within the river assemblies, 38,960,389 bases were attributed to the novel Pakistani breeds (Azikheli 1, Azikheli 2, Nili-Ravi 1, and Nili-Ravi 2).

Consistent with good-quality variant calling, the transitions to transversions (Ti/Tv) ratio observed in our graph genome was 2.17, and comparable to the Ti/Tv ratio for whole-genome sequencing (WGS) data of *B. taurus* and *B. bubalus* in a previous study [15].

### Genetic variation observed across global water buffalo populations

**Figure 2:**
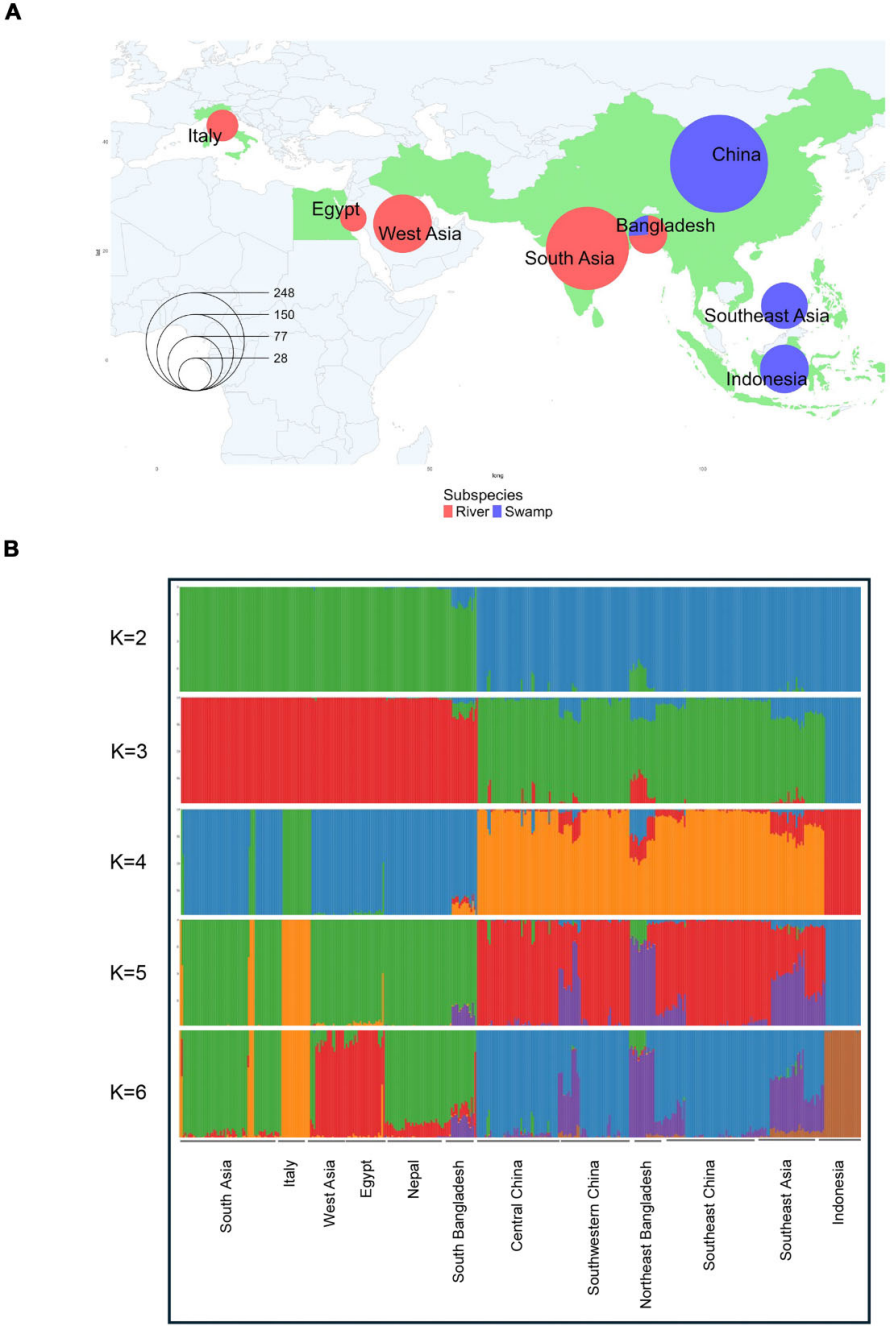
(A) Geographic distribution of global water buffalo populations used in the study. The size of each pie corresponds to the relative sample size, while red and blue colors represent river and swamp buffalo subspecies, respectively. (B) Admixture plot for different K values ranging from K = 2 to K = 6.

We next sought to examine the frequency and segregation patterns of the variants identified in our pangenome across wider water buffalo populations. To do this, we obtained WGS data from 8 bioprojects (PRJNA633724, PRJEB39591, PRJNA547460, PRJCA001294, PRJNA350833, PRJNA1135737, PRJNA1057008, PRJNA633919) totaling 937 individuals. After filtering on depth of sequencing and sequencing approach, 711 were kept for downstream analyses, comprising 374 swamp and 337 river buffalo. The geographic distribution and admixture levels of these samples are shown in Fig. 2 (PCA plots shown in Supplementary Fig. S4). The metadata details of the WGS cohort are provided in Supplementary Table S1.

Variants found in our newly generated reference graph genome (water buffalo pangenome) were explicitly genotyped in each sample using PanGenie. Following stringent filtering for variants with a missing genotype rate >0.20 and minor allele frequency <0.05, 27,369,548 of 32,821,175 variants were retained for subsequent analysis.

For PCA and admixture analyses, these samples were further filtered to remove closely related samples, leaving 403 individuals (175 river and 228 swamp buffalo), and following LD pruning, 678,504 biallelic SNVs were retained. As expected, genetic differentiation broadly reflects geography. The first principal component (PC1), accounting for 81.04% of the variance (Supplementary Fig. S4), as well as the admixture analysis at K = 2, effectively separated the 2 buffalo subspecies, river and swamp (Fig. 2B). Some evidence of admixture between the 2 subspecies is observed in the hybrid zone in Bangladesh, consistent with previous studies [16].

This PanGenie-genotyped cohort consequently provides a globally representative collection of water buffalo variant calls, both spanning the largest number of samples to date (711 individuals) and for the first time incorporating both short (SNVs) and longer variants (SVs). To enable reuse, we have made this dataset of chromosome-specific VCF files available at [53].

### Concordance of graph and linear reference calls

**Figure 3:**
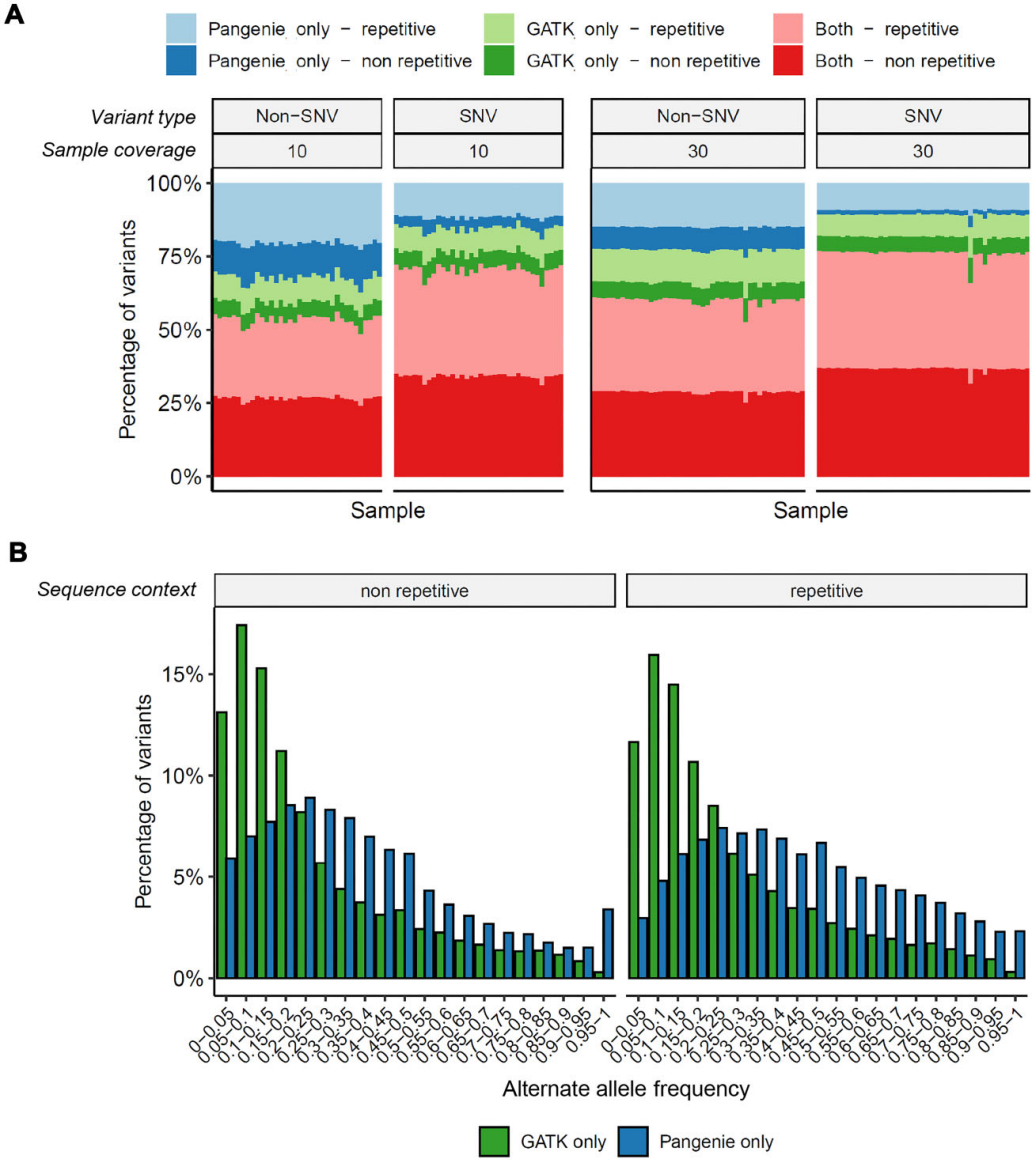
(A) Agreement in genotype calls from GATK and PanGenie across 81 river buffalo samples. In each plot, each column corresponds to a sample, and the y-axis indicates the proportion of variants called by both variant callers (red) or only by PanGenie (blue) or GATK (green). Intensity of color indicates the variants’ sequence context (found in repetitive or nonrepetitive sequence contexts). Panels are further broken down by variant type (SNV or non-SNV) and the approximate coverage of the samples (10× or 30× sequencing coverage). (B) The allele frequencies among the samples of variants specifically called only by either GATK or PanGenie in the high-coverage (30×) samples. The results are broken down according to whether variants are found in repetitive regions.

Before undertaking downstream analyses with this dataset, we wanted to address the open question of the relative advantages and disadvantages of graph-based versus single reference-based variant calling in water buffalo research. To begin to address this, we examined the concordance of the PanGenie-derived genotyping calls in a cohort of 81 samples to those derived from the traditional variant caller GATK. As shown in Fig. 3A, for each higher coverage sample, approximately 77% of SNVs were called by both callers, with the remaining variants relatively evenly split between those specific to GATK (13%) and those specific to PanGenie (10%). The majority (83%) of SNVs in nonrepetitive regions were called by both genotypers, with few SNV calls in nonrepetitive regions being specific to PanGenie (3.9%). This is consistent with GATK being better able to detect SNVs, especially in less complex genomic regions, in part because PanGenie is dependent on variants being present in the set of analyzed assemblies to be genotyped. For non-SNVs, such as insertions and deletions, a higher proportion of variants were specific to PanGenie: 26% and 19% in repetitive and nonrepetitive regions, respectively. In comparison, GATK only called an extra 20% and 14%, respectively. These results are broadly consistent with the idea that graph callers are potentially better able to detect non-SNV calls than traditional genotyping tools. However, an important disadvantage of callers such as PanGenie is their limitation to only calling genotypes at variants represented in the genome graph. This is emphasized when examining the allele frequency of variants specific to one or the other caller (Fig. 3B). GATK-specific calls are comparatively enriched with those with a low allele frequency. This is consistent with PanGenie missing these rarer variants due to their lower frequency and therefore their lower probability of being represented in the graph. No substantial difference in the proportion of variants out of Hardy–Weinberg equilibrium was observed between the sets of variants specific to each caller (Supplementary Fig. S5). However, a difference in Ti/Tv ratios was observed, with the PanGenie-specific variant calls generally having a lower ratio (Supplementary Fig. S6), associated with putatively more false positives. This likely in part reflects that the GATK calls were filtered based on metric cutoffs guided by Ti/Tv ratios [15]. On average the Ti/Tv ratio of the 711 PanGenie calls in each individual was 2.18 (Supplementary Table S4). Notably, this is higher than what was observed in the original PanGenie study, where a Ti/Tv ratio of around 2.01 was observed for human assemblies [31]. Consequently, the optimum variant caller will likely depend on the planned downstream analyses. Analyses such as the study of selective sweeps or genome-wide association studies where low-frequency variants are often filtered out will benefit less from the advantages of GATK, particularly given its longer runtime. However, studies in which it is necessary to detect private or low-frequency variants and reduce false-positive SNV rates (e.g., the study of mutation rates) will be at a disadvantage if graph-based callers such as PanGenie are used.

### Selective sweeps in water buffalo

**Figure 4:**
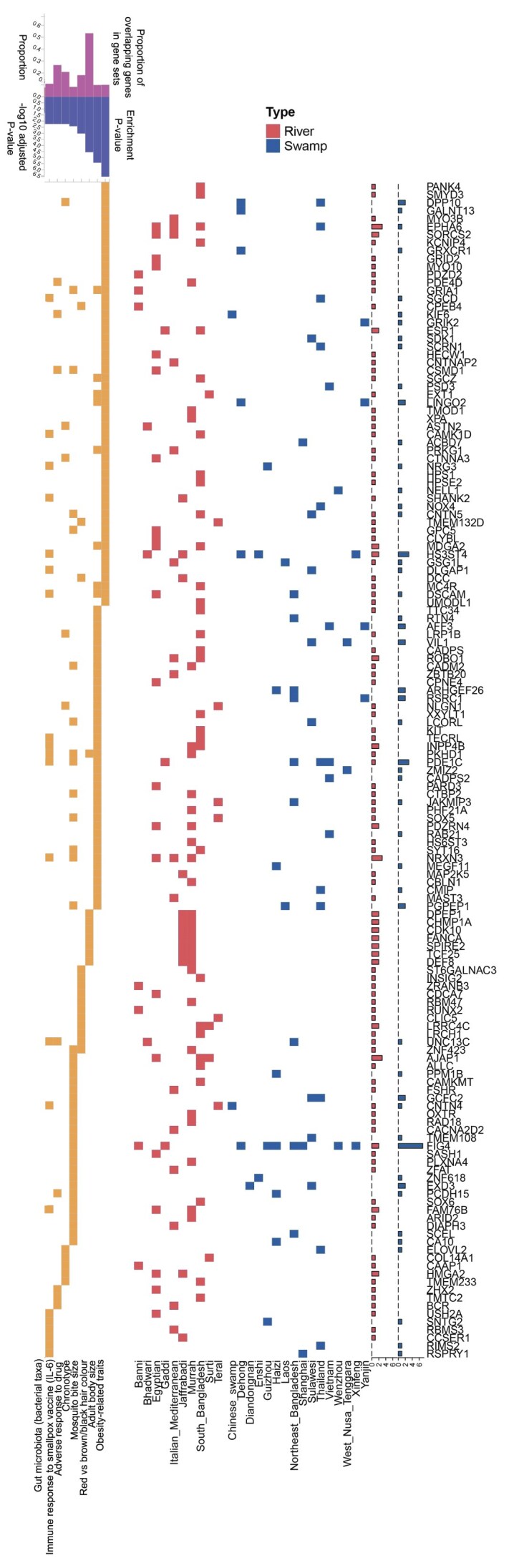
Enrichment analysis of the genes under peaks identified in the iHS analysis. The x-axis of the first panel shows human GWAS traits enriched among the genes falling under iHS peaks (as identified by FUMA), and the x-axis of the second panel shows breeds in which the corresponding selective sweeps are observed. The y-axis lists the genes within the respective gene sets and peaks, with the boxes indicating their association with specific traits and selective sweeps in various breeds. Enrichment *P* values and adjusted *P* -values (expressed as −log₁₀ values) are shown at the top left to indicate the enrichment of terms (only the top 8 most significant terms are shown). The bar graphs on the left show the total number of breeds in which a putative selective sweep peak that intersected the corresponding gene was observed.

We next explored the utility of graph genomics approaches to inform the identification of functional genes and variants under selection in water buffalo. To do this, we restricted our larger cohort to populations with at least 6 unrelated individuals. This resulted in a set of 282 samples spanning 26 distinct populations (Supplementary Table S2), consisting of 15 swamp buffalo and 10 river buffalo groups. The iHS and nSL statistics were then calculated within each group to identify sites of potential positive selection (Supplementary Table S5). In total, 1,960 genes were detected under a putative selective sweep peak for 1 or the other metric, with 249 genes identified by both (1,065 only detected by iHS and 646 by nSL; Supplementary Table S6). To explore the significance of these genes, we conducted gene set enrichment analyses using FUMA [54], focusing on enrichment among genes linked to traits in human genome-wide association studies (GWAS), due to the comparative sparsity of buffalo and livestock gene-to-trait annotations. Intriguingly, a range of relevant phenotypes were preferentially associated with the genes under putative selective sweep peaks (Fig. 4). For iHS, these ranged from obesity-related traits and adult body size to coat color and immune-relevant phenotypes, such as mosquito bite size. Furthermore, nSL highlighted additional behavioral phenotypes, from anxiety and stress-related disorders to dental health indicators, such as smooth-surface caries (Supplementary Table S7). These results consequently provide insights into the target phenotypes and underlying genes under selection in water buffalo. All population-level iHS and nSL scores can be viewed along the genome alongside other annotations, including XP-EHH and XP-CLR scores from a previous study [15] at our Bovine Omics Atlas browser (BOmA [55]; see Supplementary Fig. S7 for an example view), enabling exploration of selection signals across water buffalo populations.

More specifically, 53 genes were associated with the “obesity-related traits” term (*P* = 6.09 × 10^−11^; adjusted *P* = 2.69 × 10^−7^) in the iHS analysis, including *MC4R* in South Bangladeshi river buffalo, mutations of which are the commonest form of monogenic obesity in humans [56]. Likewise *LCORL*, under putative selection in Sulawesi swamp buffalo and among the 46 genes linked to body size, has been linked to birth weight and growth in various cattle studies [57, 58], suggesting this gene has been targeted by domestication across bovids. Intriguingly, 14 genes were linked to the “mosquito bite size” term and 6 to the “immune response to smallpox vaccine” term (Fig. 4), suggesting both artificial selection for production traits as well as natural selection for immune traits are key drivers of selective sweeps across the water buffalo populations.

### Identifying candidate larger variants linked to selective sweeps

**Figure 5:**
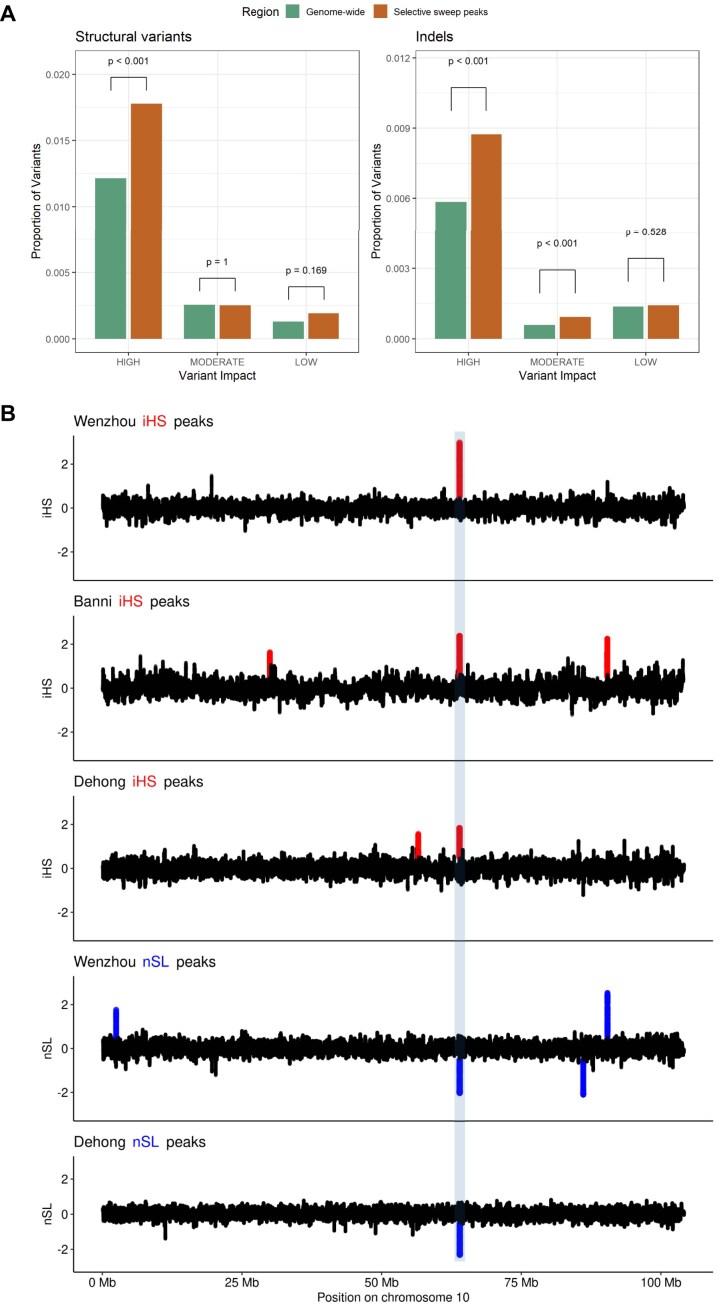
(A) Enrichment of high-impact SVs and indels in selective sweep peaks. The y-axis shows the proportion of variants in each category (genome-wide or selective sweep peaks) that are in each impact class (HIGH, MODERATE, and LOW). Due to the disproportionate size of their bars, the MODIFIER class is not shown. Two-sided Fisher exact *P* values are shown above the bars of the difference between categories of the proportions of variants in the corresponding impact class. (B) Example colocalization of selective sweep peaks observed across populations and metrics at the *FIG4* locus. Called peaks are indicated by red (iHS) or blue (nSL) points, with the respective buffalo population indicated above the plot.

A key advantage of graph genomics approaches is the ability to assay larger variants that may drive variation in phenotypes and traits, but that may have been missed in traditional approaches focused on SNPs. To investigate putative adaptive variants affecting coding regions, we annotated variants using SnpEff v5.2f [50] and SnpSift v5.2f [59]. Prior to annotation, multiallelic variants were normalized by splitting them into separate biallelic entries, resulting in 6,159,686 indels, 28,669,966 SNVs, and 160,921 SVs. Within putative selective sweep regions, we identified 208,862 indels, 997,500 SNVs, and 6,748 SVs. Notably, an enrichment of HIGH-impact SVs, indels, and SNVs was observed within selective sweep regions (Fig. 5A, Supplementary Table S8), with 50–80% more variants in these areas having a HIGH impact compared to genome-wide. Among the high-impact variants in selective sweep regions, only 20% were SNVs, with the remainder being SVs and indels, suggesting high-impact larger variants may underlie putative selective sweeps.

In total, 965 genes were affected by HIGH-impact variants falling in a putative selective sweep peak. Among the indels and large structural variants with a predicted HIGH or MODERATE impact, several also exhibited pronounced differences in aAFs between populations consistent with putative selection, including those linked to production, fertility, immunity, or adaptability traits. The detailed summary of genes associated with HIGH consequences is provided in Supplementary Table S9. As some populations in this study were comparatively small, we primarily focused on investigating in more detail selective sweep peaks detected across multiple populations.

Evidence for a strong selective sweep signal was observed exclusively in river buffalo populations at the *PGRMC2* (progesterone receptor membrane component 2) locus on chromosome 17 (Supplementary Figs. S7, S8). The *PGRMC2* gene is associated with fertility and production-related traits in bovines [60] and is additionally expressed in bovine mammary tissues during lactation in dairy cattle [61, 62].

A potential HIGH-impact 11-bp insertion was observed within the coding region of this gene at position 17:43,563,676. This insertion occurs at cDNA position c.294_295 in transcript XM_006,067,309.2, resulting in a frameshift starting at amino acid position 99. This frameshift introduces a premature stop codon downstream, disrupting the C-terminal cytoplasmic domain of *PGRMC2* [63]. Consistent with the observed difference in selection between the subspecies, the average aAF of this variant was 91% in swamp buffalo, compared to the river buffalo populations, which exhibited an average alternate allele frequency of 32%. Given the uterus’s pivotal role as a target for progesterone (P4) responses, disruptions in *PGRMC2* function are potentially likely to impair uterine function and fertility [64]. This is corroborated by epidemiological studies in humans [63, 65, 66] and livestock [63, 67], as well as genetic research in rodents [63, 66], which link low conception rates to inadequate progesterone levels and subsequent uterine dysfunction. In cattle, a similar region on chromosome BTA17 (spanning 29–34 Mb) is also strongly associated with milk fatty acid composition [62].

Selective sweeps spanning 2 neighboring genes, non-SMC conodensin I complex subunit G (*NCAPG*) and ligand-dependent nuclear receptor corepressor-like (*LCORL*), were identified on chromosome 7 (Supplementary Fig. S9). *NCAPG* encodes a subunit of the condensing 1 protein involved in chromatin condensation during replication and found to be associated in modulating fetal growth in cattle [68, 69]. *LCORL* is thought to be a transcription factor that may function during spermatogenesis in the testes and may have associated roles in height, growth, and withers in equines [69, 70]. Numerous studies have shown genetic variation at the *LCORL–NCAPG* locus is strongly associated with body size and growth traits in beef cattle [58]. In our study, these genes were found to be under strong selective sweep in Sulawesi swamp buffalo (Supplementary Fig. S9). The *LCORL* gene contains a MODERATE impact deletion, resulting in the loss of 4 alanine residues (p.Ala21_Ala23del), within exon 1 of the gene. This particular variant has the highest alternate allele frequency in the Sulawesi swamp population (AF of 0.91) consistent with selection in this group. Importantly, variation in this gene has been linked to production. Perhaps most notably, an *LCORL* frameshift variant has been linked to a range of production and morphology traits in cattle [58].

A large 14-kb HIGH-impact deletion, detected at position 10:63,987,503–64,002,148 and predicted to lead to a transcript ablation of the *FIG4* gene, fell under a selective sweep peak detected across a large number of swamp buffalo genomes (Fig. 5B). *FIG4* is crucial for neuronal and muscular functions via the phosphoinositide signaling pathways [71].

A mucin-3A-like gene (LOC102408548) falls under a selective sweep peak detected in the Shanghai, Northeast Bangladesh, Thailand, and Haizi buffalo populations (Supplementary Fig. S10). Within this gene, we detected a MODERATE-impact disruptive in-frame insertion of 390 bp specific to swamp buffalo, for which the reference allele is fixed across river buffalo populations. In contrast, this insertion shows a strikingly elevated alternate allele frequency in the corresponding Shanghai (frequency of 1), Northeast Bangladesh (0.5), Haizi (0.68), and Thailand (0.70) buffalo populations. Mucin-3A (*MUC3A*) is an epithelial glycoprotein found along the mucosal lining and plays a protective role against infectious agents and particles by providing lubrication and maintaining integrity [72]. The elevated allele frequency in relevant buffalo populations underscores the potential relevance of this SV.

These findings highlight these genes as example prime candidates for further investigation into their role in buffalo adaptation and resilience, with potential implications for conservation and selective breeding programs.

## Discussion

In this study, we present the first high-quality genome assemblies for Pakistani water buffalo breeds, integrate these assemblies into a comprehensive water buffalo pangenome, perform the largest assessment of global water buffalo variation (including structural variants) to date, and use these data to explore how positive selection targeting larger variation may be driving important buffalo phenotypes. We have made these data publicly available, including via a genome browser [55], enabling users to browse the selective sweep data across the genome and relative to other annotations.

Our newly generated Pakistani assemblies rank among the most contiguous water buffalo genomes available and are the most contiguous for river buffalo in terms of contig N50. In this study, we chose to focus on creating dual assemblies (i.e., 2 pseudo-haplotype genomes per animal) due to our primary focus on assaying structural variants. Although many previous studies have produced collapsed assemblies (i.e., 1 genome for an individual), this has the disadvantage of reducing the number of variants that can be detected. For example, at best, only 1 allele can be integrated at heterozygote sites. However, using our publicly released data, it would be possible to produce collapsed assemblies with even higher metrics, with contig N50s of 90.6 Mb (Azikheli) and 83.6 Mb (Nili-Ravi) expected, and consequently comparable to the Philippine swamp buffalo assembly, the currently most contiguous collapsed assembly.

One metric on which our assemblies rank lower is scaffold N50, largely because we chose not to invest resources in scaffolding. Given that graph pangenomes focus on aligning orthologous contigs across assemblies and identifying shared or unique sequences, extensive scaffolding offers comparatively little added value for pangenome construction. Future studies, though, may benefit from reference-based scaffolding of these assemblies to, for example, assign contigs to chromosomes.

Our integrated pangenome revealed over 140 Mb of nonredundant, nonreference paths. Although fewer swamp buffalo assemblies were included, these contributed approximately the same amount of novel sequence as the river buffalo assemblies. This likely partly reflects the fact that the reference genome used is river-derived, so more of the river-specific variation is already captured. Additionally, most structural variants appear to be subspecies-specific, consistent with limited introgression—only at the Bangladesh interface is there evidence of any appreciable gene flow between river and swamp buffalo lineages.

One potential hurdle to implementing water buffalo pangenomics has been the divergent karyotypes (2n = 50 vs. 2n = 48). Our approach, in which we split swamp buffalo chromosome 1 at its fusion point to align with the river buffalo karyotype, illustrates a straightforward solution to incorporate both subspecies into the same pangenome. Future studies may benefit from tools that automate such chromosomal splits, allowing for broader applications of graph genomics across diverse buffalo populations.

A comparison of GATK [46] (reference-based) and PanGenie [31] (graph-based) genotyping highlights the complementary strengths and limitations of each. Graph-based caller approaches are especially well suited to identifying structural variants—one of the central aims of this work—while reference-based methods, such as GATK or DeepVariant [73], frequently excel in *de novo* detection of novel SNPs and often produce fewer lower-frequency false positives. In contrast, tools such as PanGenie can only genotype variants detected in the original set of assemblies. This means that rarer variants, not found in these original samples, cannot be detected, but on the other hand, larger variants that were detected can be explicitly genotyped that would otherwise be potentially missed or miscalled by linear genome callers. In practice, the optimal choice of method thus depends on specific research goals: if discovery of large, functionally important variants is paramount, graph-based approaches may prove particularly advantageous; for high-confidence, short variant calls, traditional workflows remain valuable. In certain cases, combing both shorter variant calls from single reference methods and larger calls from graph-based methods may be optimal.

Applying selective sweep analyses to one of the largest water buffalo genomic datasets assembled so far enabled us to pinpoint hundreds of genes putatively under positive selection. Over 200 genes were identified by both of the statistical methods adopted, underscoring the robustness of these signals. Notably, these genes were frequently enriched for traits related to growth, size, and immune response—phenotypes likely under strong natural and artificial selection in water buffalo. Crucially, although the ability to initially detect selective sweep regions will likely not differ substantially between graph- and linear-based approaches when using haplotype homozygosity-based statistics, we did manage to identify multiple candidate functional larger variants in these regions, which may go undetected by single reference-based, SNP-centric approaches. These findings not only highlight the potential of graph-based genomics for discovering new selection signals but also open avenues to integrate such structural variants into breeding programs aimed at enhancing productivity, disease resistance, and other economically important traits in water buffalo.

Although our focus in this study was characterizing the potential relevance of larger variants to selective sweeps, the variant call set from this study would have utility to a diverse range of other projects, from acting as a reference panel, enabling the imputation of both short and long variants, to studying the occurrence of compound heterozygote loss-of-function variants.

Overall, our work provides important novel resources and insights into how graph genomics can accelerate our understanding of structural variation and its role in driving phenotypic diversity across a species characterized by multiple karyotypes and a complex domestication history.

## Additional Files

**Supplementary Fig. S1**. The Nili-Ravi heifer from the Punjab province of Pakistan selected for the whole-genome assembly.

**Supplementary Fig. S2**. The female Azikheli river buffalo from the Swat district of Pakistan sampled for the whole-genome assembly.

**Supplementary Fig. S3**. The cross-validation error plot depicting CV values across different values of K, with K = 6 highlighted with the lowest CV error.

**Supplementary Fig. S4**. Principal component analysis (PCA) plot using the graph genome as a reference reveals clear geographical clustering both between and within the river and swamp buffalo populations.

**Supplementary Fig. S5**. The distribution of Hardy–Weinberg equilibrium (HWE) *P* values of variants specifically called by GATK (green) or PanGenie (blue). Variants are broken down into those falling or not falling into repetitive regions, and the genome-wide significance threshold of 5 × 10^–8^ is indicated by gray dashed lines. Neither variant caller shows a large number of variants above this threshold.

**Supplementary Fig. S6**. The distributions of per sample transition/transversion ratios of the SNVs called by both variant callers (red) or by only GATK (green) or PanGenie (blue). Results are broken down by the approximate coverage of the samples and whether the variant falls within a repetitive region.

**Supplementary Fig. S7**. An example view in our BOmA genome browser of the *PGRMC2* locus with evidence of a selective sweep overlapping a coding insertion marked by the vertical dashed line.

**Supplementary Fig. S8**. The genome-wide selective sweep peaks within the *PGRMC2* gene at the chromosome 17 locus, identified by iHS analysis, revealed a pronounced selective sweep signal in river buffalo populations but not in swamp buffalo populations.

**Supplementary Fig. S9**. The putative Sulawesi selective sweep spanning 2 candidate genes *NCAPG* and *LCORL* on chromosome 7.

**Supplementary Fig. S10**. The prominent selective peaks within the *MUC3A* gene, specific to the swamp buffalo population.

**Supplementary Table S1**. The data accession numbers and relevant information for each whole-genome sequencing (WGS) biosample used to capture genetic variation across global water buffalo populations.

**Supplementary Table S2**. The details of biosample accessions and grouping information of population groups selected for the selective sweeps analysis, in which the population with at least 6 unrelated individuals per group was included. The final column indicates the 282 samples used in the selective sweep analysis.

**Supplementary Table S3**. Length of paths not present in the reference genome by chromosome.

**Supplementary Table S4**. Summary statistics of PanGenie genotyped WGS cohort.

**Supplementary Table S5**. Locations of the putative selective sweep peaks by breed and metric.

**Supplementary Table S6**. The list of genes identified in potential selective sweeps, categorizing those exclusive to the iHS and nSL metrics as well as those identified by both of these.

**Supplementary Table S7**. The list of FUMA enrichment analysis of genes involved in selective sweeps identified by the iHS and nSL metric, detailing gene sets, associated trait terms, and *P* values.

**Supplementary Table S8**. The counts of HIGH, MODERATE, LOW, and MODIFIER impact variants, categorized into SNVs, indels, and large SVs, across genome-wide autosomal regions and selective sweep regions.

**Supplementary Table S9**. The list of genes along with descriptions of their roles that are impacted by HIGH-impact variant consequences within regions identified as undergoing selection.

giaf099_Supplemental_Files

giaf099_Authors_Response_To_Reviewer_Comments_Original_Submission

giaf099_GIGA-D-25-00171_Original_Submission

giaf099_GIGA-D-25-00171_Revision_1

giaf099_Reviewer_1_Report_Original_Submission Paul Stothard -- 6/3/2025

giaf099_Reviewer_2_Report_Original_Submission Yi Zhang -- /4/2025

giaf099_Reviewer_3_Report_Original_Submission Laura Caquelin -- 6/9/2025

giaf099_Reviewer_4_Report_Original_Submission Wai Yee Low -- 6/9/2025

## Abbreviations

aAF: alternate allele frequency; BUSCO: Benchmarking Universal Single-Copy Orthologs; CNGB: China National Gene Bank; CV: cross-validation; ENA: European Nucleotide Archive; GWAS: genome-wide association studies; iHS: integrated haplotype score; LMIC: low- and middle-income country; NCBI: National Center for Biotechnology Information; NGDC: National Genomics Data Centre; NIBGE: National Institute for Biotechnology and Genetic Engineering; nSL: number of segregating sites by length; PAF: pairwise alignment format; PBS: phosphate-buffered saline; PCA: principal component analysis; SNP: single-nucleotide polymorphism; SNV: single-nucleotide variant; SV: structural variant; Ti/Tv: transition/transversion; VCF: variant call file; WBC: white blood cell; WGS: whole-genome sequencing.

## Data Availability

The PacBio HiFi sequencing data generated in this study, including raw FASTQ reads and haplotype-resolved genome assemblies, have been deposited in the European Nucleotide Archive (ENA) under bioProject accession PRJEB86148. The associated raw FASTQ reads are available under sample accessions SAMEA117759395 (SRA: ERR14792573) and SAMEA117759394 (SRA: ERR14792572) for Nili-Ravi (NR0003) and Azikheli (AZ0004) buffalo, respectively. The individual haplotype resolved genome assemblies are accessible under the corresponding assembly accessions, including GCA_965246665 (Azikheli 1), GCA_965642195 (Azikheli 2), GCA_965642205 (Nili-Ravi1), and GCA_965642185 (Nili-Ravi2). The publicly available genome assemblies used in the study, including UOA_WB_1 (GCF_003121395.1), NDDB_DH_1(GCA_019923925.1), NDDB_SH_1 (GCF_019923935.1), and PCC_UOA_SB_1v2 (GCF_029407905.1), were retrieved from NCBI, and CUSA_SWP (GWHAAJZ00000000) and CUSA_RVB (GWHAAKA00000000) were accessed from NGDC. The genome assembly Wang_2023 was downloaded from Figshare, as documented in [12]. The BBCv1.0 genome was obtained from the Sequence Archive CNSA under the project accession CNP0000797, which can be accessed at [9]. All additional supporting data are available in the *GigaScience* repository, GigaDB [74], with separate datasets for Nili-Ravi breed [75] and Azikheli breed [76].
