# Supplementary material for: A comprehensive water buffalo pangenome reveals extensive structural variation linked to population-specific signatures of selection
Source: Gigascience. 2025 Aug 30;14:giaf099. doi: 10.1093/gigascience/giaf099 (PMC12398277; doi:10.1093/gigascience/giaf099)
Supplement: giaf099_Supplemental_Files [file giaf099_supplemental_files.zip › SupplementaryFigures1to10.docx]

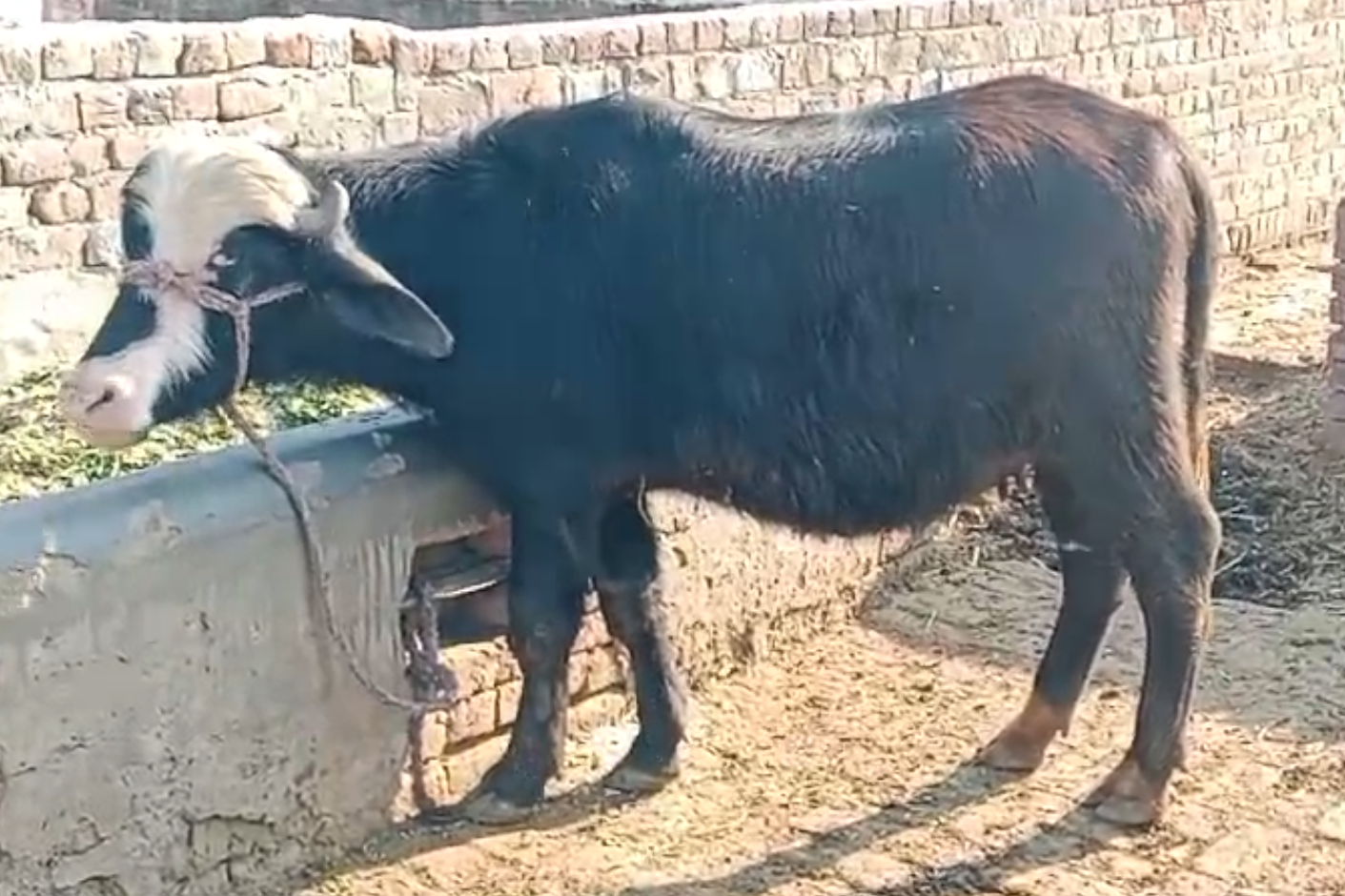


**Supplementary Fig. S1**


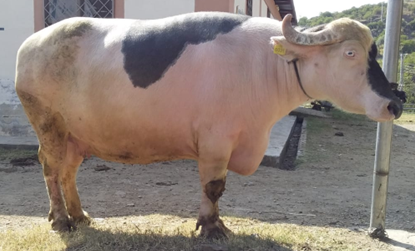


**Supplementary Fig. S2**


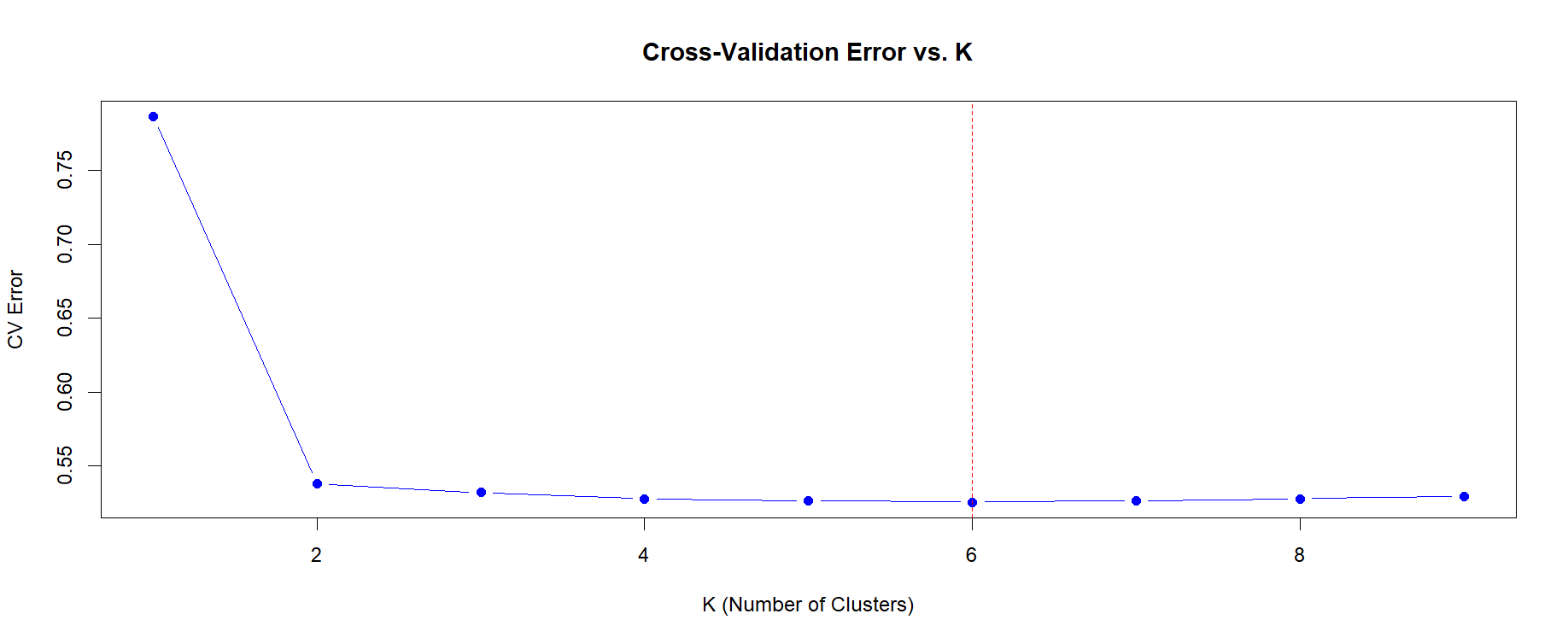


**Supplementary Fig. S3**


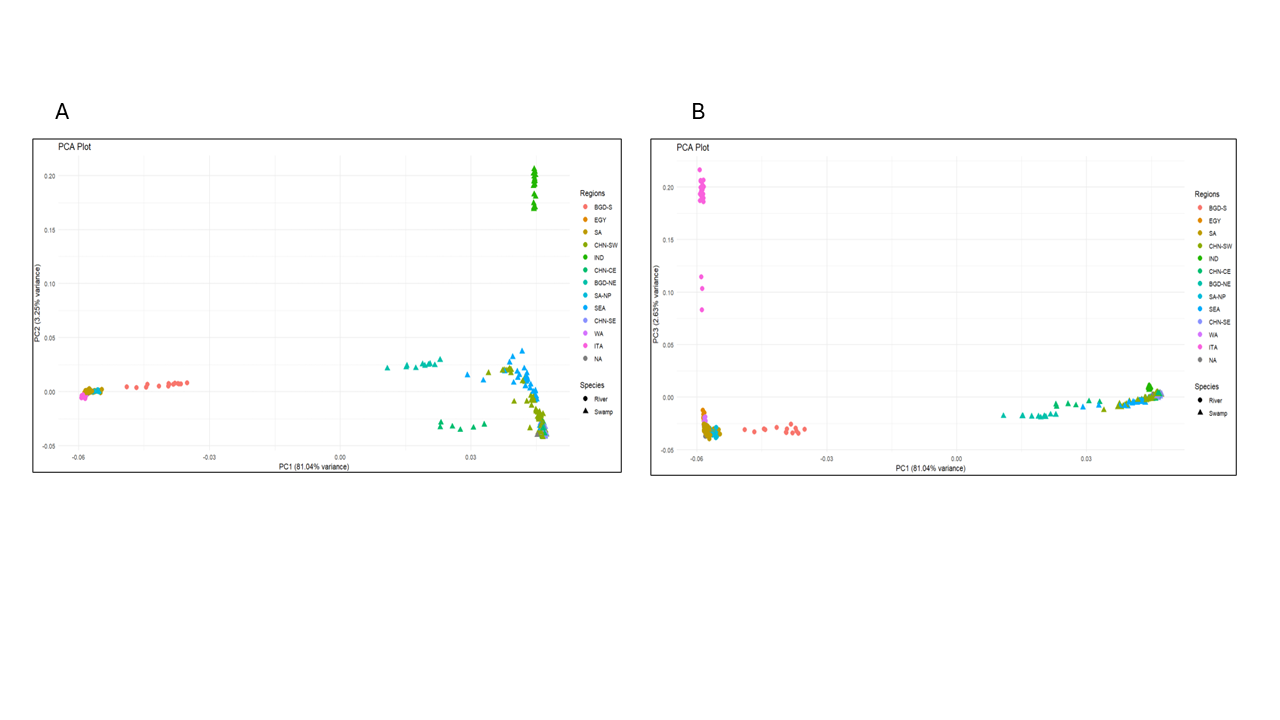


**Supplementary Fig. S4**

**
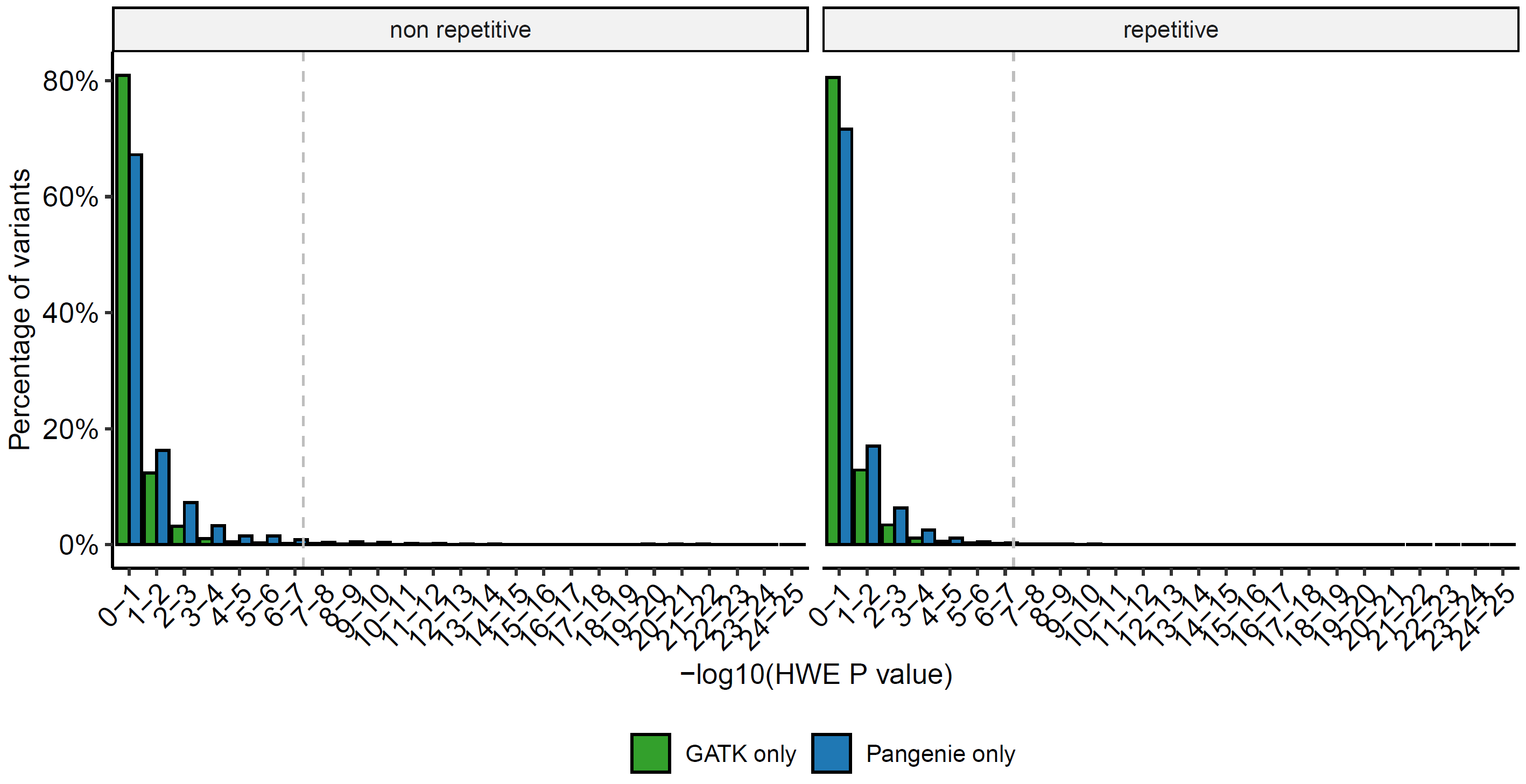
**

**Supplementary Fig. S5**

**
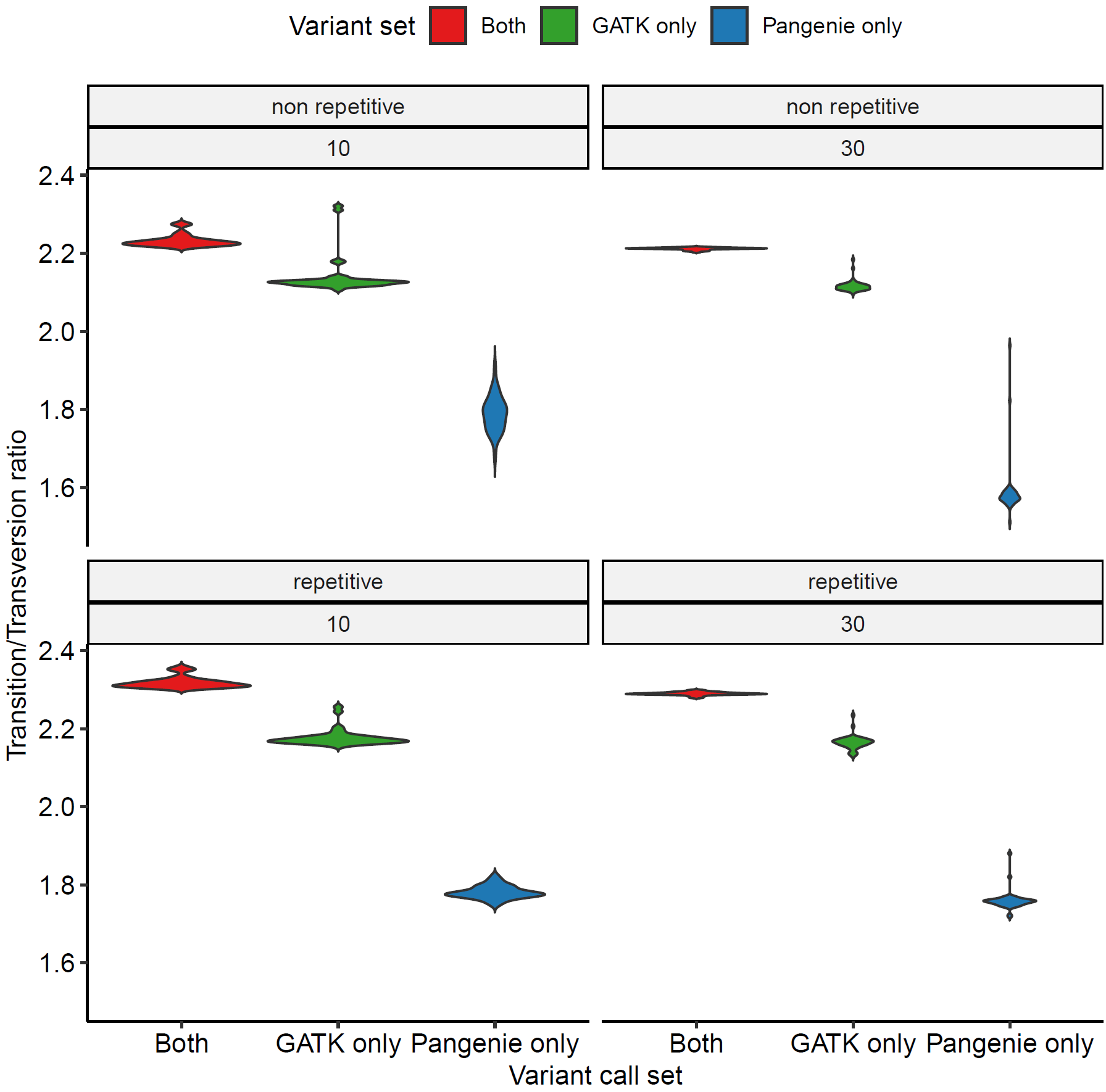
**

**Supplementary Fig. S6**

**
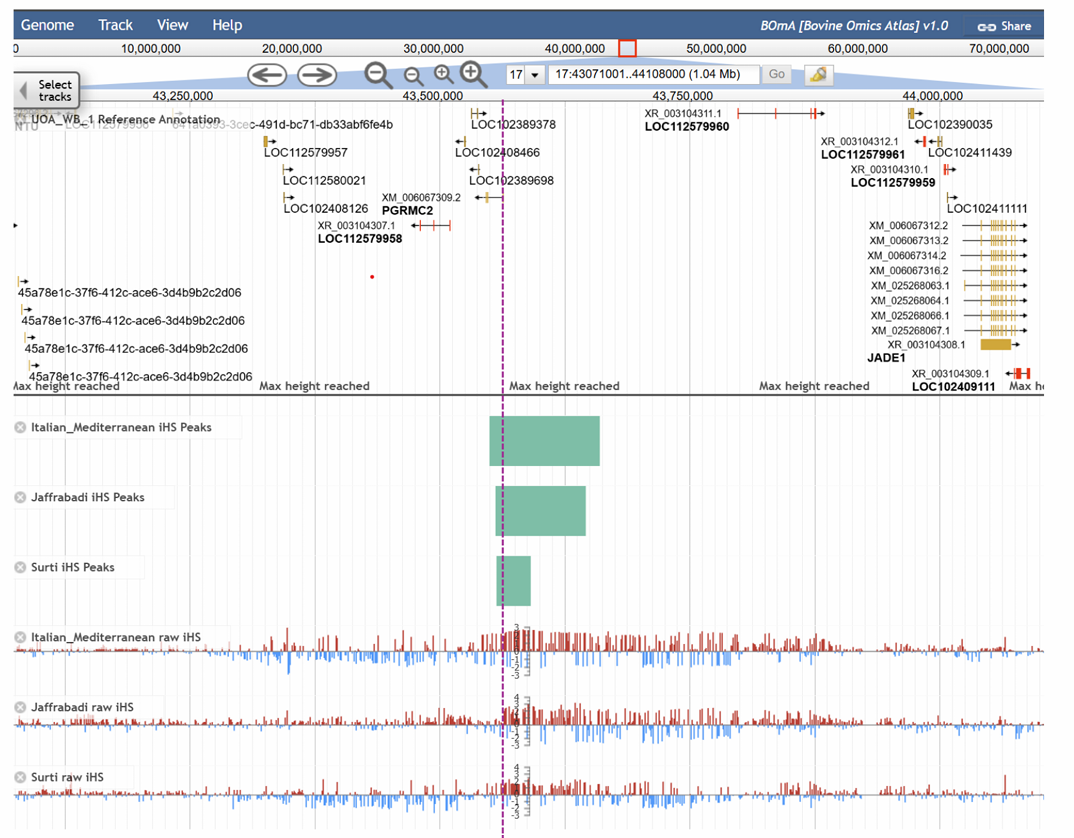
**

**Supplementary Fig. S7**

**
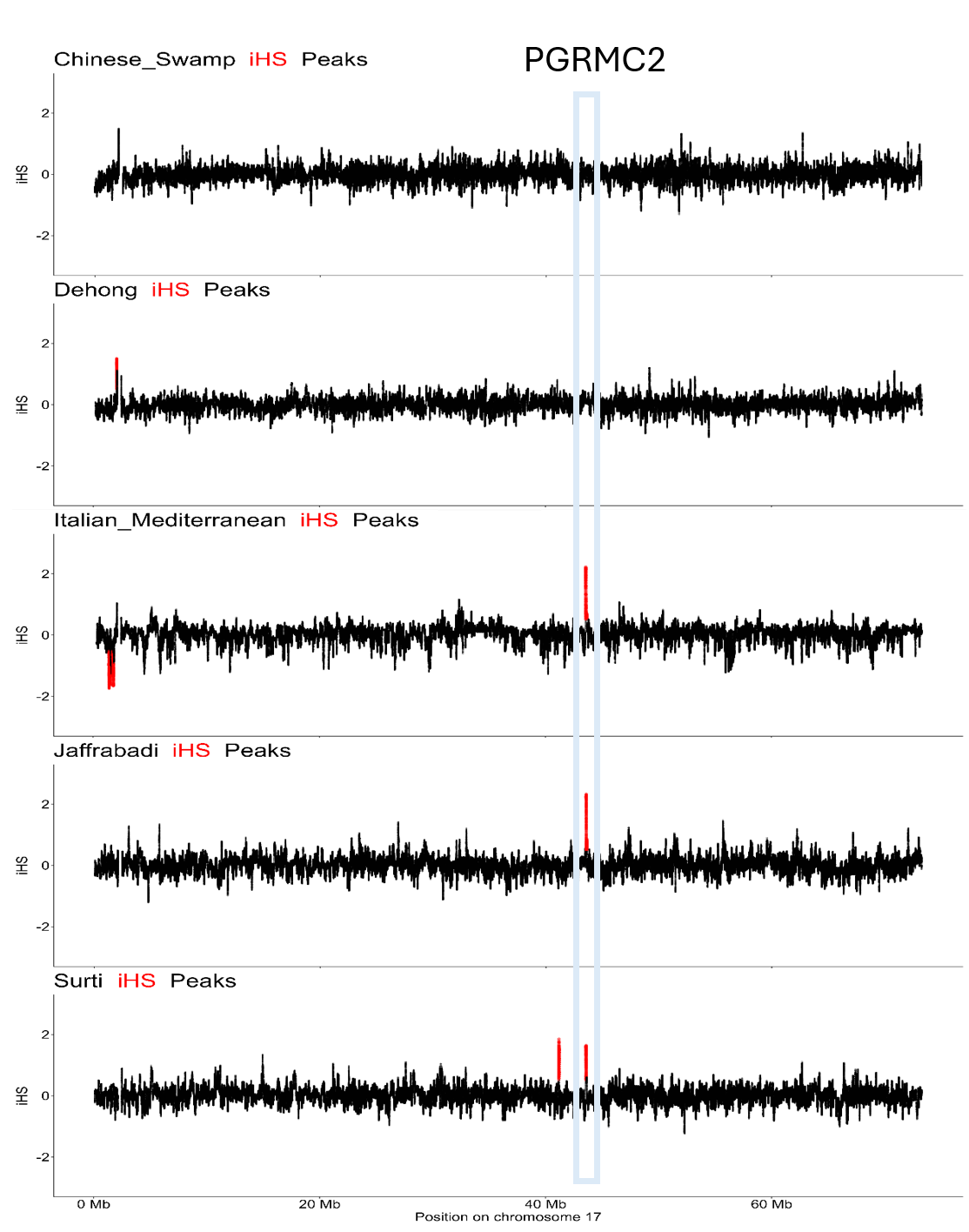
**

**Supplementary Fig. S8**

**
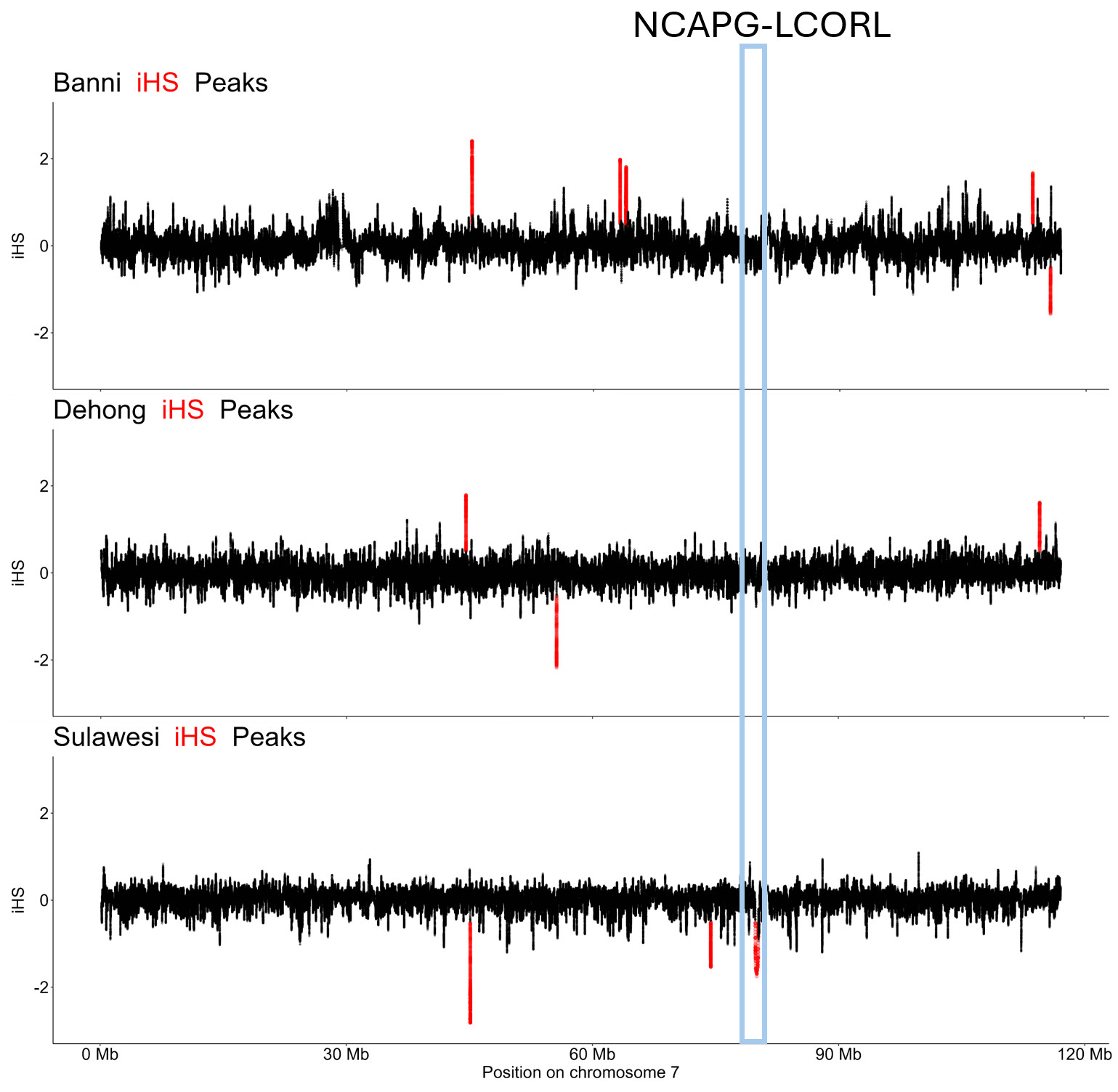
**

**Supplementary Fig. S9**

**
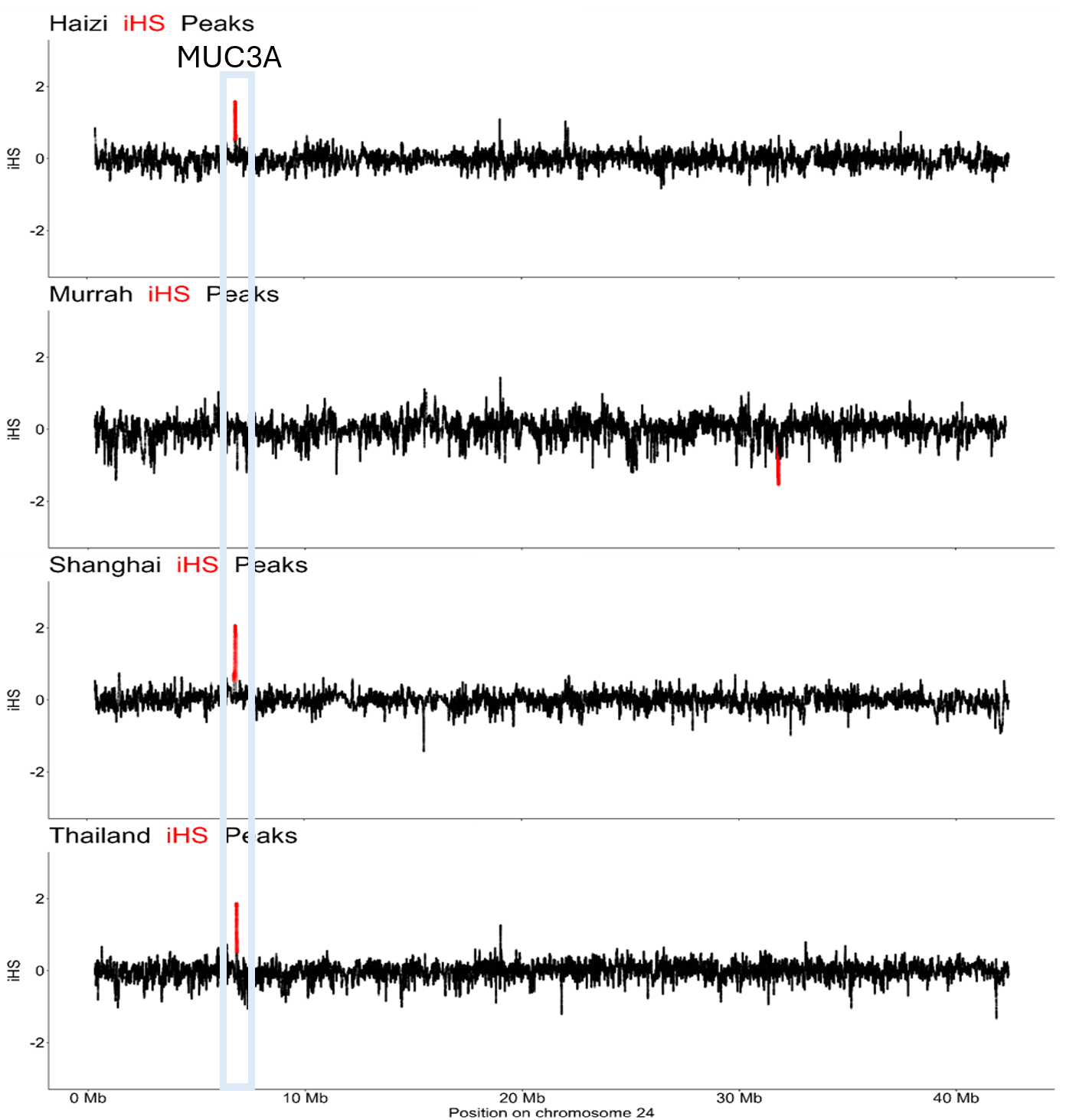
**

**Supplementary Fig. S10**
