# Supplementary material for: A comprehensive water buffalo pangenome reveals extensive structural variation linked to population-specific signatures of selection
Source: Gigascience. 2025 Aug 30;14:giaf099. doi: 10.1093/gigascience/giaf099 (PMC12398277; doi:10.1093/gigascience/giaf099)
Supplement: giaf099_Authors_Response_To_Reviewer_Comments_Original_Submission [file giaf099_authors_response_to_reviewer_comments_original_submission.pdf]

Author's Response To Reviewer Comments

Reviewer #1: This well-written manuscript describes the generation of new genome assemblies for water buffalo and the con
limitations are stated. The manuscript should be of high interest to the water buffalo research community and to those intere
I have minor comments that I believe should be addressed prior to publication.
Minor comments:
In the NCBI genomes database, the water buffalo assembly NDDB_SH_1 is listed as the current reference genome, not UOA_

Response: Thank you for noticing this. We have now clarified at (lines 184-185) that we chose UOA_WB_1 as it remains the a
and previous analyses (such as Dutta et al., enabling our comparison to the GATK calls done relative to this genome and for u

Lines 64-69: Lack of clarity regarding relationships among water buffalo populations:
- Wording suggests single domestication event accounts for all domestic water buffalo. But, the river and swamp buffalo dive
- Taxonomic terminology is inherently ambiguous for a few reasons, including:
1) The Bubalus arnee species comprises both wild river buffalo and wild swamp buffalo, which have not been assigned subspe
2) Domestic water buffalo (including river and swamp buffalo) are assigned their own species name: Bubalus bubalis, despite
3) Unlike their wild source populations, domesticated river buffalo and domesticated swamp buffalo are assigned their own sp
- To address ambiguity regarding taxonomy and phylogeny of the buffalo populations, mention the full subspecies names (Bu

Response: Many thanks. We have now rewritten the paragraph to clarify these points (lines 64-67) and now give the full trin

Line 82: "Although eight higher quality": higher quality than what?

Response: Have clarified we mean comparatively higher quality than others available (line 84-85).

Line 177: Undefined acronym: "PAF".

Response: Now spelled out as “pairwise-alignment (.paf) format” (line 189).

Line 216: "each unique biosamples": should be "each unique biosample".

Response: Corrected (line 230).

Line 272: Which SnpEff database was used for variant annotation?

Response: Clarified gene annotation used. (line 293-295).

Line 286-287: Based on Table 1, the difference between the largest and the smallest water buffalo genome is 360 mega base
that segmental duplications are often not accurately represented in genome assemblies, there is a strong possibility that som
the Discussion.

Response: We agree with the reviewer that inflation in genome sizes due to assembly artefacts cannot be completely exclude
repetitive regions being better resolved and is a trend that is observed in other species and is supported by the high BUSCO c
https://www.biorxiv.org/content/10.1101/2025.04.17.649430v1.full). We now discuss this at lines 313-315.

Line 360-361: Elaborate slightly on what is in the dataset being shared.

Response: Thank you for the suggestion. We have now clarified that this dataset comprises VCF files of the genotype calls (lin

Line 420-421: Clarify which of these are human vs animal traits.

Response: These are all human GWAS traits (see second line in Figure legend).

Figure 1 A legend: The dots seem to all be the same size, which suggests that this is a scatter plot, not a bubble plot.

Response: Thanks. Legend corrected (line 321).

Figure 1 C: "across the graph genome" sounds spatial; perhaps "proportion of variant types in the graph genome" would be c

Response: Changed to “Proportion of variant types in the pangenome graph” (line 329)

Figure 1 D: It would be helpful to have the rows sorted to match the order in B.
Figure 1 D: The low bars (i.e. small number of shared sites) are not easy to interpret. Perhaps the y-axis could be transforme

Response: Figure 1D now updated accordingly.

Reviewer #2: This manuscript presents the first high-quality, haplotype-resolved genome assemblies for two representative F
non-reference sequence and 111,352 SVs. By genotyping of 711 global samples against this pangenome, the authors uncove
highlights graph genomics as a transformative tool for integrative analyses of evolutionarily related species in an unbiased wa
General Comments
1.The study's methodology is rigorous, combining long-read assembly, graph-based genotyping (PanGenie), and population-l

Response: Thanks. We do discuss limitations such as missing rare variants, for example see line 413 “This is consistent with
hurdle to implementing water buffalo pangenomics has been the divergent karyotypes”

2. The selection signature analyses were done across a number of population groups but the paper only showcases a limited s
selective signals specific to certain groups.

Response: Thanks. As the reviewer says we think looking across populations groups to identify common selective signals can
S5) listing all putative selective sweep peaks by breed for both metrics so readers can explore all regions and which of them a

3. It could be informative to conduct comparative analyses of selection signatures using variant datasets from PanGenie and

Response: The detection of the location of selective sweeps is not expected to be substantively different between the GATK a
rather than, for example, just looking at allele frequency differences at individual variants, and for this reason are often even
structural variants. We have added a discussion of this at lines 618-620.

Specific Comments
1. In Figure 1D and the main text, the rationale behind dividing the SVs into 40 sets is not clearly presented. If the interpreta

Response: Thanks, we have made updates to this Figure in the revised manuscript. There is a long tail of sets which would be

2. Lines 325 - 326 state that the newly assembled pangenome graph exhibits a substantial increase in genome size compared

Response: We have now included the extra bases in paths by chromosome in Supplementary Table S3. The extra bases is str

3. In lines 410 - 412, there may be an issue with the citation of Table S2. The table contains 402 individuals, whereas the tex

Response: Column F of Supplementary table 2 indicates which of the 282 samples were used in the selective sweep analysis.

4. Figure 3 shows that, when using 30x samples in the variant calling comparison between Pangenie and GATK, there are stil

Response: Thanks. We have expanded the text (lines 401-405) explaining that many GATK-only SNVs are rare, so are conse

5. To provide a more intuitive understanding of how SV can influence gene function and contribute to the traits, the authors c

Response: We have now included a screenshot from our BOmA browser indicating the location of the coding indel in PGRMC2

Reviewer #3: Reproducibility report for: A comprehensive water buffalo pangenome reveals extensive structural variation lin
Reviewer(s): Laura Caquelin, Department of Clinical Neuroscience, Karolinska Institutet, Sweden

- Summary of the computational reproducibility review The Fisher's exact tests for enrichment across variant and impact cate
- Recommendations for authors
We were able to reproduce study with the data and information provided in the Figure 5A description. To further improve tran
-- Make the codes to reproduce all analyses in the paper openly available to allow anyone to reproduce the results. Ideally, p
-- Include statistical outputs, such as exact p-values, in supplementary materials when possible. This ensures clarity and eas
--- The definition of each variable name.
--- The origin of each dataset (raw, processed, etc).
--- Any preprocessing steps applied before analysis.

Response: Many thanks for taking the time to check this. We have updated the code and supplementary material accordingly

Reviewer #4: Review of "A comprehensive water buffalo pangenome reveals extensive structural variation linked to populatio
Line 30: I think it is better you include the actual number of publicly available assemblies used to create the pangenome gra

Response: Added: “eight publicly available assemblies” (line 30).

Line 71: There is now a swamp buffalo reference genome with annotation too (NCBI accession: PCC_UOA_SB_1v2). Perhaps
https://eur02.safelinks.protection.outlook.com/?url=https%3A%2F%2Facademic.oup.com%2Fgigascience%2Farticle%2Fdoi
bXB0eU1hcGkiOnRydWUsIlYiOiIwLjAuMDAwMCIsIlAiOiJXaW4zMiIsIkFOIjoiTWFpbCIsIldUIjoyfQ%3D%3D%7C0%7C%7C%7C
enough.

Response: Thanks. In response also to reviewer 1 we have reworded text around reference assemblies throughout (including

Line 79: "highlighted"

Response: Corrected (line 81).

Line 82: What do you mean by "higher quality"? The assemblies have been discussed in this review:
https://eur02.safelinks.protection.outlook.com/?url=https%3A%2F%2Fwww.frontiersin.org%2Fjournals%2Fgenetics%2Fartic
U1hcGkiOnRydWUsIlYiOiIwLjAuMDAwMCIsIlAiOiJXaW4zMiIsIkFOIjoiTWFpbCIsIldUIjoyfQ%3D%3D%7C0%7C%7C%7C&sdata

Response: We have now clarified this at line 84-85.

Line 105: Technically, the graph method for bovine species, which includes water buffalo, is being investigated by the Bovine
(https://eur02.safelinks.protection.outlook.com/?url=https%3A%2F%2Fgenomebiology.biomedcentral.com%2Farticles%2F1
0&data=05%7C02%7C%7Ceef2951ee5174877164d08dda764bddb%7C2e9f06b016694589878910a06934dc61%7C0%7C0%
cAE2V6OpU%3D&reserved=0).

Response: Added citation (line 102).

Line 165: It will be good if you add a bit more context of the PanGenie method here as the researchers in buffalo community

Response: We have expanded on the approach adopted by PanGenie (lines 179-182); code accompanies the manuscript.

Line 170: To produce phase pangenome graph, don't you need all input assemblies to be phased? All are input assemblies ph

Response: Apologies for the confusion. We had meant the variants are called per assembly so their calls are effectively phase

Line 235: "a list of 403 unrelated individuals." What does this translate to in terms that geneticists can understand? Do you n

Response: Rewritten to: “kin pairs closer than third-degree (KING kinship > 0.0625) were pruned” (lines 245-246).

Line 246: Can you please explain how did you get the coordinates to match between the GATK and PanGenie method? You'll

Response: We confirm both call-sets are against UOA_WB_1 with the Pangenie callset also relative to this assembly (e.g. see

Line 254: Why these 3 chromosomes?

Response: We selected 2, 12, 22 to sample large, medium and small autosomes; now stated explicitly (line 273).

Line 257: If you had not filtered for relatedness, how will it impact the selective sweep work? I think including some context [illegible]

Response: Sentence added noting that including close kin inflates long-range haplotype statistics and can create false-positiv[illegible]

Line 259: do you mean at least six samples per group? If yes, is 6 samples enough?

Response: Yes, six samples i.e. 12 haplotypes. An advantage of tests such as the integrated haplotype score is that it estima[illegible] observed across the whole genome in the set of samples studied by calculating a z score. Effectively this means that both fal[illegible] identified in smaller groups. Importantly we primarily focused on peaks seen across populations, effectively increasing the se[illegible]

Line 261: genotype quality less than 25 according to bcftools? Since you only used biallelic variants, please provide the break[illegible]

Response: Yes GQ from Pangenie output but was filtered using bcftools. Numbers added: 31,504,264 biallelic, 1,316,911 mu[illegible]

Line 281: "… we first PacBio HiFi sequenced one female" Please rewrite this.

Response: Reworded at line 304.

Line 282: How common are these two breeds in percentage?

Response: We have updated this text at line 306.

Line 291: Is this already known? Perhaps cite the literature to show the agreement with previous studies?

Response: We have now cited a previous study of global animals using short read data (line 319).

Fig 1D: This is a bit too small to see especially the SV distribution at the bottom. I can hardly see the median?

Response: This figure has now been revised accordingly.

Line 310: Why did you choose UOA_WB_1 as the reference

Response: See response to first comment of reviewer 1 above.

Line 311: the ~32.8 mil variants are comprised of SNPs as well?

Response: Yes this is all variants. The numbers by type follow in the text (lines 339-342).

Fig 2: This is probably a panel of a figure but should not be the entire figure. The size of the circle indicates sample size but t[illegible]
Line 356: S Figure 4 and 5 should be main figures? You will need to annotate the abbreviation of sample-country in the legen[illegible]

Response: Thanks for the suggestions. Supplementary Figure 5 is now moved to be panel B in Figure 2. Panel A has been up[illegible]

Line 360: "To enable reuse we have made this dataset available …" The dataset should be made available to reviewers?

Response: This is now available (https://zenodo.org/records/15741377).

Line 368: "76% of SNVs were called by both callers" 76% seem low. Also, called does not mean concordant. What is the conc[illegible]

Response: Apologies for the confusion. Here we did effectively mean concordance, though the tool we used, vcfeval, scores n[illegible] assemblies used to create the graph. As most variants are rare, and thus often missing from these assemblies, they will natu[illegible] specific calls are largely restricted to repetitive regions and longer variants, where assemblies generated from long reads hav[illegible]

Fig 3B: It is not immediately clear what the difference is, between non repetitive and repetitive regions. The overlapping text

Response: Apologies, we noticed an issue with our analysis of repetitive regions where we were using the start and end of the
This change though has a limited impact on Figure 3B because there simply isn't much of a difference between repetitive and

Line 390: "Analyses such as the study of selective sweeps or genome-wide association studies where low frequency variants

Response: Thanks for the comment. We believed a sentence was required to highlight how Pangenie calls could be effective f
clarification here if possible. We though leave going into more detail of the relative merits of linear reference and graph base

Line 418: Why human? Could you use cattle?

Response: High quality gene-set annotations for livestock phenotypes are unfortunately comparatively sparse; whereas huma

Line 427: I tried the browser and not sure what I can learn from it. It will be helpful if there is a README with some example

Response: We have now included an example screenshot as supplementary Figure S7 illustrating how the browser can be use

Line 450: How large before you considered it as larger variant? Is this ability to study larger variants still hold despite using
reads. As such, the selective sweep may not be associated with any larger variants?

Response: Although some studies use 50bp as a threshold to define SVs this cutoff has no biological basis to it. Likewise the
traditional linear approaches, but are not proscribing a given length for these as there isn't a simple cutoff where one approa
The number of assemblies does impact the ability to define variants (as rarer ones wont be detected) but it has a comparativ
larger variants detected in the graph are genotyped in the short read data and assayed in the selective sweep analysis (thoug

Line 470: Fig S8 should be a main figure?

Response: If ok our preference would be to leave this as a supplementary figure as we have already provided one of these pl

Line 513: Instead of uniprot link, perhaps consider including this as Supplementary info or text. The info in the link may char

Response: URL removed.

Line 551: However, without scaffolding, the assemblies of Pakistani river buffalo may not be good enough to function as refer

Response: We have now discussed this at line 583-585.

Line 552: When considering new bases, did you do this for each assembly independently or the new bases were discovered c

Response: Was done by looking at alternative paths in the non-redundant graph, so not each assembly independently. Clarifi

Line 581: Some of my questions at Line 450 can be discussed here.

Response: See response above.

Line 586: Perhaps consider discussing the limitations of the small number of assemblies used to create the graph. As such, m

Response: We discuss the fact that rarer variants will be missing from the graph at lines 403-405. However, we are able to a
an arbitrary cutoff and filter by size in this study.

Editorial Manager®

Cookie Preference Center

We use cookies which are necessary to make our site work. We may also use additional cookies to analyze, improve and personalize our content and your digital experience. For more information, see our Cookie Policy.

You may choose not to allow some types of cookies. However, blocking some types may impact your experience of our site and the services we are able to offer. See the different category headings below to find out more or change your settings.

Allow all

Manage Consent Preferences

Strictly Necessary Cookies

Always active

These cookies are necessary for the website to function and cannot be switched off in our systems. They are usually only set in response to actions made by you which amount to a request for services, such as setting your privacy preferences, logging in or filling in forms. You can set your browser to block or alert you about these cookies, but some parts of the site will not then work.

Functional Cookies

These cookies enable the website to provide enhanced functionality and personalisation. They may be set by us or by third party providers whose services we have added to our pages. If you do not allow these cookies then some or all of these services may not function properly.

Performance Cookies

These cookies allow us to count visits and traffic sources so we can measure and improve the performance of our site. They help us to know which pages are the most and least popular and see how visitors move around the site.

Targeting Cookies

These cookies may be set through our site by our advertising partners. They may be used by those companies to build a profile of your interests and show you relevant adverts on other sites. If you do not allow these cookies, you will experience less targeted advertising.

Cookie List

Clear

Apply Cancel
Consent Leg.Interest

Confirm my choices

Powered by onetrust
