# Supplementary material for: A comprehensive water buffalo pangenome reveals extensive structural variation linked to population-specific signatures of selection
Source: Gigascience. 2025 Aug 30;14:giaf099. doi: 10.1093/gigascience/giaf099 (PMC12398277; doi:10.1093/gigascience/giaf099)
Supplement: giaf099_Reviewer_1_Report_Original_Submission — Paul Stothard -- 6/3/2025 [file giaf099_reviewer_1_report_original_submission.pdf]

Reviewer Report

Title: A comprehensive water buffalo pangenome reveals extensive structural variation linked to population specific signatures of selection

Version: Original Submission **Date:** 6/3/2025

Reviewer name: Paul Stothard

Reviewer Comments to Author:

This well-written manuscript describes the generation of new genome assemblies for water buffalo and the construction of a pangenome graph that is used for variant calling and downstream analyses. The work is clearly described and the methods are appropriate given the goals of the study. The results are interesting and timely, and realistic limitations are stated. The manuscript should be of high interest to the water buffalo research community and to those interested in applying pangenome graphs to variant calling.
I have minor comments that I believe should be addressed prior to publication.
Minor comments:
In the NCBI genomes database, the water buffalo assembly NDDB_SH_1 is listed as the current reference genome, not UOA_WB_1 as suggested in the manuscript. Perhaps the reference genome was recently reassigned?
Lines 64-69: Lack of clarity regarding relationships among water buffalo populations:
- Wording suggests single domestication event accounts for all domestic water buffalo. But, the river and swamp buffalo diverged prior to the domestication date. This is a contradiction.
Clarify by mentioning that there were at least two independent domestication events (one for river buffalo and one for swamp buffalo).
- Taxonomic terminology is inherently ambiguous for a few reasons, including:
1) The Bubalus arnee species comprises both wild river buffalo and wild swamp buffalo, which have not been assigned subspecies names.
2) Domestic water buffalo (including river and swamp buffalo) are assigned their own species name: Bubalus bubalis, despite being biologically the same species as Bubalus arnee.
3) Unlike their wild source populations, domesticated river buffalo and domesticated swamp buffalo are assigned their own species names, Bubalus bubalis bubalis and Bubalus bubalis carabanensis, respectively.
- To address ambiguity regarding taxonomy and phylogeny of the buffalo populations, mention the full subspecies names (Bubalus bubalis bubalis, and Bubalus bubalis carabanensis).
Line 82: "Although eight higher quality": higher quality than what?

Line 177: Undefined acronym: "PAF".
Line 216: "each unique biosamples": should be "each unique biosample".
Line 272: Which SnpEff database was used for variant annotation?
Line 286-287: Based on Table 1, the difference between the largest and the smallest water buffalo genome is 360 mega base pairs. That exceeds the length of the largest chromosome by almost 2 fold, and is 14% of the total length of the UOA_WB_1 reference assembly. This is a very large difference to observe between members of the same species. Considering that segmental duplications are often not accurately represented in genome assemblies, there is a strong possibility that some of the variants identified between these new high-quality assemblies and the other assemblies are simply assembly artefacts (failure of recently duplicated segments to be distinguished, etc.). At the very least, this should be addressed in the Discussion.
Line 360-361: Elaborate slightly on what is in the dataset being shared.
Line 420-421: Clarify which of these are human vs animal traits.
Figure 1 A legend: The dots seem to all be the same size, which suggests that this is a scatter plot, not a bubble plot.
Figure 1 C: "across the graph genome" sounds spatial; perhaps "proportion of variant types in the graph genome" would be clearer.
Figure 1 D: It would be helpful to have the rows sorted to match the order in B.
Figure 1 D: The low bars (i.e. small number of shared sites) are not easy to interpret. Perhaps the y-axis could be transformed to log scale or the number of variants could be added to the bars.

Methods

Are the methods appropriate to the aims of the study, are they well described, and are necessary controls included? Choose an item.

Conclusions

Are the conclusions adequately supported by the data shown? Choose an item.

Reporting Standards

Does the manuscript adhere to the journal's guidelines on minimum standards of reporting? Choose an item.

Choose an item.

Statistics

Are you able to assess all statistics in the manuscript, including the appropriateness of statistical tests used? Choose an item.

Quality of Written English

Please indicate the quality of language in the manuscript: Choose an item.

Declaration of Competing Interests

Please complete a declaration of competing interests, considering the following questions:

- Have you in the past five years received reimbursements, fees, funding, or salary from an organisation that may in any way gain or lose financially from the publication of this manuscript, either now or in the future?
- Do you hold any stocks or shares in an organisation that may in any way gain or lose financially from the publication of this manuscript, either now or in the future?
- Do you hold or are you currently applying for any patents relating to the content of the manuscript?
- Have you received reimbursements, fees, funding, or salary from an organization that holds or has applied for patents relating to the content of the manuscript?
- Do you have any other financial competing interests?
- Do you have any non-financial competing interests in relation to this paper?

If you can answer no to all of the above, write 'I declare that I have no competing interests' below. If your reply is yes to any, please give details below.

I declare that I have no competing interests

I agree to the open peer review policy of the journal. I understand that my name will be included on my report to the authors and, if the manuscript is accepted for publication, my named report including any attachments I upload will be posted on the website along with the authors' responses. I agree for my report to be made available under an Open Access Creative Commons CC-BY license (http://creativecommons.org/licenses/by/4.0/). I understand that any comments which I do not wish to be included in my named report can be included as confidential comments to the editors, which will not be published.

Choose an item.

To further support our reviewers, we have joined with Publons, where you can gain additional credit to further highlight your hard work (see: https://publons.com/journal/530/gigascience). On publication of this paper, your review will be automatically added to Publons, you can then choose whether or not to claim your Publons credit. I understand this statement.

Yes Choose an item.
