# Supplementary material for: A comprehensive water buffalo pangenome reveals extensive structural variation linked to population-specific signatures of selection
Source: Gigascience. 2025 Aug 30;14:giaf099. doi: 10.1093/gigascience/giaf099 (PMC12398277; doi:10.1093/gigascience/giaf099)
Supplement: giaf099_Reviewer_2_Report_Original_Submission — Yi Zhang -- /4/2025 [file giaf099_reviewer_2_report_original_submission.pdf]

Reviewer Report

Title: A comprehensive water buffalo pangenome reveals extensive structural variation linked to population specific signatures of selection

Version: Original Submission **Date:** 6/4/2025

Reviewer name: Yi Zhang

Reviewer Comments to Author:

This manuscript presents the first high-quality, haplotype-resolved genome assemblies for two representative Pakistani river buffalo breeds (Nili Ravi and Azikheli), integrating them with existing assemblies to construct a water buffalo pangenome. The study leverages graph genomics to characterize structural variation (SV), identifying >140 Mb of non-reference sequence and 111,352 SVs. By genotyping of 711 global samples against this pangenome, the authors uncover population-specific selective sweeps linked to productivity, immunity, and adaptation traits, revealing potentially functional SVs, though these findings are limited by the absence of validation evidence and cross-study comparisons. The work highlights graph genomics as a transformative tool for integrative analyses of evolutionarily related species in an unbiased way and provides resources to accelerate buffalo breeding.

General Comments

1.The study's methodology is rigorous, combining long-read assembly, graph-based genotyping (PanGenie), and population-level sweep scans. Nevertheless, the manuscript would benefit from discussion of graph limitations, such as bias against rare variants (Fig. 3B) and challenges in graph construction for species with karyotypic divergence.

2. The selection signature analyses were done across a number of population groups but the paper only showcases a limited selection of results. To strengthen the manuscript, the authors could concentrate on a consistent set of populations. This would enable a more in-depth examination of selective signals common across buffalo population groups or unique selective signals specific to certain groups.

3. It could be informative to conduct comparative analyses of selection signatures using variant datasets from PanGenie and GATK. This could reveal whether the pangenome approach might uncover important structural variants within selection signals that GATK fails to identify.

Specific Comments

1. In Figure 1D and the main text, the rationale behind dividing the SVs into 40 sets is not clearly presented. If the interpretation is correct, the y-axis label of the bar graph should denote the number of SVs rather than size. Moreover, the main title "SVs Size Distribution" at the top seems more relevant to the box plots at the bottom.

2. Lines 325 - 326 state that the newly assembled pangenome graph exhibits a substantial increase in genome size compared to the existing reference genome. It is recommended that the authors describe the distribution of the 147,865,364 bp across the entire genome. Are they found more prevalent in specific regions of certain chromosomes?

3. In lines 410 - 412, there may be an issue with the citation of Table S2. The table contains 402 individuals, whereas the text mentions 282.

4. Figure 3 shows that, when using 30x samples in the variant calling comparison between Pangenie and GATK, there are still a large number of SNV variants detectable only by GATK. A more in-depth technical discussion of these differences would greatly enhance the reader's comprehension of these findings and the relative performance of the two methods.
5. To provide a more intuitive understanding of how SV can influence gene function and contribute to the traits, the authors could include a figure that displays an example gene structure along with the SV of interest within a selection signal peak.

Methods

Are the methods appropriate to the aims of the study, are they well described, and are necessary controls included? Choose an item.

Conclusions

Are the conclusions adequately supported by the data shown? Choose an item.

Reporting Standards

Does the manuscript adhere to the journal's guidelines on minimum standards of reporting? Choose an item.

Choose an item.

Statistics

Are you able to assess all statistics in the manuscript, including the appropriateness of statistical tests used? Choose an item.

Quality of Written English

Please indicate the quality of language in the manuscript: Choose an item.

Declaration of Competing Interests

Please complete a declaration of competing interests, considering the following questions:

- Have you in the past five years received reimbursements, fees, funding, or salary from an organisation that may in any way gain or lose financially from the publication of this manuscript, either now or in the future?
- Do you hold any stocks or shares in an organisation that may in any way gain or lose financially from the publication of this manuscript, either now or in the future?
- Do you hold or are you currently applying for any patents relating to the content of the manuscript?

- Have you received reimbursements, fees, funding, or salary from an organization that holds or has applied for patents relating to the content of the manuscript?
- Do you have any other financial competing interests?
- Do you have any non-financial competing interests in relation to this paper?

If you can answer no to all of the above, write 'I declare that I have no competing interests' below. If your reply is yes to any, please give details below.

I declare that I have no competing interests

I agree to the open peer review policy of the journal. I understand that my name will be included on my report to the authors and, if the manuscript is accepted for publication, my named report including any attachments I upload will be posted on the website along with the authors' responses. I agree for my report to be made available under an Open Access Creative Commons CC-BY license (http://creativecommons.org/licenses/by/4.0/). I understand that any comments which I do not wish to be included in my named report can be included as confidential comments to the editors, which will not be published.

Choose an item.

To further support our reviewers, we have joined with Publons, where you can gain additional credit to further highlight your hard work (see: https://publons.com/journal/530/gigascience). On publication of this paper, your review will be automatically added to Publons, you can then choose whether or not to claim your Publons credit. I understand this statement.

Yes Choose an item.
