# Supplementary material for: A comprehensive water buffalo pangenome reveals extensive structural variation linked to population-specific signatures of selection
Source: Gigascience. 2025 Aug 30;14:giaf099. doi: 10.1093/gigascience/giaf099 (PMC12398277; doi:10.1093/gigascience/giaf099)
Supplement: giaf099_Reviewer_3_Report_Original_Submission — Laura Caquelin -- 6/9/2025 [file giaf099_reviewer_3_report_original_submission.pdf]

Reviewer Report

Title: A comprehensive water buffalo pangenome reveals extensive structural variation linked to population specific signatures of selection

Version: Original Submission **Date:** 6/9/2025

Reviewer name: Laura Caquelin

Reviewer Comments to Author:

Reproducibility report for: A comprehensive water buffalo pangenome reveals extensive structural variation linked to population specific signatures of selection Journal: Gigascience
ID number/DOI: GIGA-D-25-00171
Reviewer(s): Laura Caquelin, Department of Clinical Neuroscience, Karolinska Institutet, Sweden
----
1. SummaryoftheStudy
This study used graph genomics to better characterize water buffalo genomes. By building a pangenome from new and existing assemblies, the authors analyzed 711 samples. These samples revealed structural variation. These results highlight the value of graph genomics. This method ---
2. Scopeofreproducibility
According to our assessment the primary objective is: to identify genomic variants within selective sweep regions in the water buffalo genome.
- Outcome: Enrichment of high-impact structural variants (SVs), insertions/deletions (indels) and single nucleotide variants (SNVs) in selective sweep regions.
- Analysis method outcome: Variants were compared between selective sweep regions and genome-wide. Fisher's exact test was used to assess enrichment of functional variants.
- Main result: "Prior to annotation, multiallelic variants were normalized by splitting them into separate biallelic entries, resulting in 6,159,686 indels, 28,669,966 SNVs, and 160,921 SVs entries. Within putative selective sweep regions we identified 208,862 indels, 997,500 SNVs and 6,748 SVs. Notably an enrichment of HIGH impact SVs, indels and SNVs were observed within selective sweep regions (Figure 5A, Supplementary Table S6), with 50-80% more variants in these areas having a HIGH impact compared to genome-wide. Among the high impact variants in selective sweep regions only 20% were SNVs, with the remainder being SVs and indels, suggesting high impact larger variants may underlie putative selective sweeps." (Lines 453 to 461)
----
3. AvailabilityofMaterials a. Data
- Data availability: Open
- Data completeness: Complete, all data necessary to reproduce main results are available
- Access Method: Supplementary files - Repository: -
- Data quality: Structured b. Code
- Code availability: Shared for the review after request - Programming Language(s): R

- Repository link: -
- License: -
- Repository status: -
- Documentation: No documentation

----

4. Computational environment of reproduction analysis
- Operating system for reproduction: MacOS 14.7.4
- Programming Language(s): R
- Code implementation approach: Creating script according to the methodology description/Using shared code
- Version environment for reproduction: R version 4.4.1/RStudio 2024.09.0

----

5. Results
5.1 Original study results
- Results 1: Results are presented in Figure 5A.
5.2 Steps for reproduction
-> Reproduce the results
The code was not shared initially, but as the data were provided and the test was a Fisher's exact test, I wrote code to reproduce the p-values.
- Issue 1: P-values for the SNVs variant as well as the Â« Modifier Â» impact class were not provided.
-- Resolved: Authors provided an updated Supplementary table S6 with exact numerical p-values for each variant and each impact class. The code "variantEnrichAtPeaks.R" to generate the Figure 5A and the Supplementary table S6 was also shared.
New version of the supplementary Table S6: (see screenshot)
The comparison between the reproduced results and the original results was then performed using the shared code. (Notably, the results from the R script written allowed for the generation of the same p-value as the one presented in Figure 5A).
- Issue 2: In the script "variantEnrichAtPeaks.R", only the figures were generated, not the new supplementary Table S6 with the numerical p-values.
-- Resolved: Some code lines was added in the function "makePlot" to generate this table in addition to the figure.
Line 159 to 178 of the script "variantEnrichAtPeaks_RCC."

```
##########################################
## 5. Supplementary table S6 (add) ##
##########################################
summary_table <- df %>%
mutate(
Type = variantType,
Genome_Wide_Prop = Genome_wide / sum(Genome_wide), Selective_Sweep_peaks_Prop = Sweep / sum(Sweep), Ratio_of_proportions = Selective_Sweep_peaks_Prop /
Genome_Wide_Prop) %>%
left_join(pval_df, by = "Impact") %>%
```

```
select(
Impact, Type,
Genome_Wide = Genome_wide,
`Selective_Sweep peaks` = Sweep,
`Genome_Wide Prop` = Genome_Wide_Prop,
`Selective_Sweep peaks Prop`= Selective_Sweep_peaks_Prop,
`Ratio of proportions`= Ratio_of_proportions,
`Fishers exact P` = p_value)
return(list(plot = p,
summary_table = summary_table))
########################################
```

5.3 Statistical comparison Original vs Reproduced results

- Results: Figure and table S6 were reproduced for each variant type and impact:
-- SVs type: (see screenshot)
-- Indels type: (see screenshot)
-- And SNVs type: (see screenshot)
- Comments: The shared code was used to compute the p-values and generated the Figures. Minor numerical error discrepancy was observed for some p-values, likely due to rounding differences. The p-values in the original Excel file appear to be stored with less decimal precision than those computed in R. This difference is negligible and does not indicate a reproducibility issue.
- Errors detected: No error detected.
- Statistical Consistency: The results were successfully reproduced with the share code.

----

6. Conclusion

- Summary of the computational reproducibility review

The Fisher's exact tests for enrichment across variant and impact categories, presented in Figure 5A of the manuscript, were successfully reproduced using the data in supplementary table S6 and the shared code. Results were consistent with the original, with only negligible rounding differences in p-values.

- Recommendations for authors

We were able to reproduce study with the data and information provided in the Figure 5A description. To further improve transparency and ensure full reproducibility of your manuscript, the following recommendations are suggested:

-- Make the codes to reproduce all analyses in the paper openly available to allow anyone to reproduce the results. Ideally, provide a README or requirements.txt file describing how to run the analysis, including software versions, packages, and dependencies.
-- Include statistical outputs, such as exact p-values, in supplementary materials when possible. This ensures clarity and eases verification. Ideally, provide metadata: For the datasets used or generated by the scripts, it would be helpful to include accompanying metadata files that explain:
--- The definition of each variable name.
--- The origin of each dataset (raw, processed, etc).
--- Any preprocessing steps applied before analysis.

Methods

Are the methods appropriate to the aims of the study, are they well described, and are necessary controls included? Choose an item.

Conclusions

Are the conclusions adequately supported by the data shown? Choose an item.

Reporting Standards

Does the manuscript adhere to the journal's guidelines on minimum standards of reporting? Choose an item.

Choose an item.

Statistics

Are you able to assess all statistics in the manuscript, including the appropriateness of statistical tests used? Choose an item.

Quality of Written English

Please indicate the quality of language in the manuscript: Choose an item.

Declaration of Competing Interests

Please complete a declaration of competing interests, considering the following questions:

- Have you in the past five years received reimbursements, fees, funding, or salary from an organisation that may in any way gain or lose financially from the publication of this manuscript, either now or in the future?
- Do you hold any stocks or shares in an organisation that may in any way gain or lose financially from the publication of this manuscript, either now or in the future?
- Do you hold or are you currently applying for any patents relating to the content of the manuscript?
- Have you received reimbursements, fees, funding, or salary from an organization that holds or has applied for patents relating to the content of the manuscript?
- Do you have any other financial competing interests?
- Do you have any non-financial competing interests in relation to this paper?

If you can answer no to all of the above, write 'I declare that I have no competing interests' below. If your reply is yes to any, please give details below.

1.no 2.no 3.no 4.no 5.no 6.no

I agree to the open peer review policy of the journal. I understand that my name will be included on my report to the authors and, if the manuscript is accepted for publication, my named report including any attachments I upload will be posted on the website along with the authors' responses. I agree for my report to be made available under an Open Access Creative Commons CC-BY license (http://creativecommons.org/licenses/by/4.0/). I understand that any comments which I do not wish to be included in my named report can be included as confidential comments to the editors, which will not be published.

Choose an item.

To further support our reviewers, we have joined with Publons, where you can gain additional credit to further highlight your hard work (see: https://publons.com/journal/530/gigascience). On publication of this paper, your review will be automatically added to Publons, you can then choose whether or not to claim your Publons credit. I understand this statement.

Yes Choose an item.
