# Supplementary material for: A comprehensive water buffalo pangenome reveals extensive structural variation linked to population-specific signatures of selection
Source: Gigascience. 2025 Aug 30;14:giaf099. doi: 10.1093/gigascience/giaf099 (PMC12398277; doi:10.1093/gigascience/giaf099)
Supplement: giaf099_Reviewer_4_Report_Original_Submission — Wai Yee Low -- 6/9/2025 [file giaf099_reviewer_4_report_original_submission.pdf]

Reviewer Report

Title: A comprehensive water buffalo pangenome reveals extensive structural variation linked to population specific signatures of selection

Version: Original Submission **Date:** 6/9/2025

Reviewer name: Wai Yee Low

Reviewer Comments to Author:

Review of "A comprehensive water buffalo pangenome reveals extensive structural variation linked to population specific signatures of selection". This is an impressive work at the frontier of buffalo genomics. I truly enjoy reading the work and my questions/comments are aimed at improving it further. My detailed comments are below:
Line 30: I think it is better you include the actual number of publicly available assemblies used to create the pangenome graph.
Line 71: There is now a swamp buffalo reference genome with annotation too (NCBI accession: PCC_UOA_SB_1v2). Perhaps consider to cite the swamp buffalo ref https://academic.oup.com/gigascience/article/doi/10.1093/gigascience/giae053/7753516 and rewrite the sentence to say a pangenome can be used for both swamp and river, but a single linear ref from either subspecies for read mapping is not good enough.
Line 79: "highlighted"
Line 82: What do you mean by "higher quality"? The assemblies have been discussed in this review: https://www.frontiersin.org/journals/genetics/articles/10.3389/fgene.2021.629861/full
Line 105: Technically, the graph method for bovine species, which includes water buffalo, is being investigated by the Bovine Pangenome Consortium (BPC). However, nothing useful has been published on the buffalo graph but perhaps consider citing the BPC since your paper overlaps with it (https://genomebiology.biomedcentral.com/articles/10.1186/s13059-023-02975-0).
Line 165: It will be good if you add a bit more context of the PanGenie method here as the researchers in buffalo community are not used to this. Additionally, it will be great if all code is made available on GitHub or as Supplementary Info.
Line 170: To produce phase pangenome graph, don't you need all input assemblies to be phased? All are input assemblies phased? The UOA_WB_1 is locally phased, not phased throughout the genome.
Line 235: "a list of 403 unrelated individuals." What does this translate to in terms that geneticists can understand? Do you mean siblings have been removed? Or individuals sharing the same grandparents were removed?
Line 246: Can you please explain how did you get the coordinates to match between the GATK and PanGenie method? You'll need matching coordinates for concordance analysis. As I understand it, the GATK was based on UOA_WB_1?
Line 254: Why these 3 chromosomes?
Line 257: If you had not filtered for relatedness, how will it impact the selective sweep work? I think including some context will help the readers.

Line 259: do you mean at least six samples per group? If yes, is 6 samples enough?
Line 261: genotype quality less than 25 according to bcftools? Since you only used biallelic variants, please provide the breakdown between biallelic and multiallelic.
Line 281: "... we first PacBio HiFi sequenced one female" Please rewrite this.
Line 282: How common are these two breeds in percentage?
Line 291: Is this already known? Perhaps cite the literature to show the agreement with previous studies?
Fig 1D: This is a bit too small to see especially the SV distribution at the bottom. I can hardly see the median?
Line 310: Why did you choose UOA_WB_1 as the reference?
Line 311: the ~32.8 mil variants are comprised of SNPs as well?
Fig 2: This is probably a panel of a figure but should not be the entire figure. The size of the circle indicates sample size but there should be a legend on the plot for this to say the sizes, right? Darker colour should be used to highlight the countries with samples instead of white? Maybe this could be a Supp figure too.
Line 356: S Figure 4 and 5 should be main figures? You will need to annotate the abbreviation of sample-country in the legend of S Figure 5.
Line 360: "To enable reuse we have made this dataset available ..." The dataset should be made available to reviewers?
Line 368: "76% of SNVs were called by both callers" 76% seem low. Also, called does not mean concordant. What is the concordance among called SNVs in both? Did the pangenome approach called most of the variants found in GATK? If not, what might be the reasons?
Fig 3B: It is not immediately clear what the difference is, between non repetitive and repetitive regions. The overlapping text in the x-axes makes it hard to read.
Line 390: "Analyses such as the study of selective sweeps or genome-wide association studies where low frequency variants are often filtered out will benefit less from the advantages of GATK, particularly given its longer run time." From here on, in this paragraph, it's Discussion, not Results.
Line 418: Why human? Could you use cattle?
Line 427: I tried the browser and not sure what I can learn from it. It will be helpful if there is a README with some examples on what can be explored.
Line 450: How large before you considered it as larger variant? Is this ability to study larger variants still hold despite using only ~10 assemblies in the graph? The use of short reads for selective sweep study will still benefit from being able to incorporate these larger variants? As I understand it, the larger variants were found only from graph, not from the short reads. As such, the selective sweep may not be associated with any larger variants?
Line 470: Fig S8 should be a main figure?
Line 513: Instead of uniprot link, perhaps consider including this as Supplementary info or text. The info in the link may change in the future.
Line 551: However, without scaffolding, the assemblies of Pakistani river buffalo may not be good enough to function as reference genomes for river buffalo?
Line 552: When considering new bases, did you do this for each assembly independently or the new bases were discovered cumulatively?

Line 581: Some of my questions at Line 450 can be discussed here.
Line 586: Perhaps consider discussing the limitations of the small number of assemblies used to create the graph. As such, many SVs are likely still missing and we are still unable to properly assess allele frequency of these larger SVs. Additionally, while some SVs may not be considered as large in this work, it does not mean they have no impact.

Methods

Are the methods appropriate to the aims of the study, are they well described, and are necessary controls included? Choose an item.

Conclusions

Are the conclusions adequately supported by the data shown? Choose an item.

Reporting Standards

Does the manuscript adhere to the journal's guidelines on minimum standards of reporting? Choose an item.

Choose an item.

Statistics

Are you able to assess all statistics in the manuscript, including the appropriateness of statistical tests used? Choose an item.

Quality of Written English

Please indicate the quality of language in the manuscript: Choose an item.

Declaration of Competing Interests

Please complete a declaration of competing interests, considering the following questions:

- Have you in the past five years received reimbursements, fees, funding, or salary from an organisation that may in any way gain or lose financially from the publication of this manuscript, either now or in the future?
- Do you hold any stocks or shares in an organisation that may in any way gain or lose financially from the publication of this manuscript, either now or in the future?
- Do you hold or are you currently applying for any patents relating to the content of the manuscript?
- Have you received reimbursements, fees, funding, or salary from an organization that holds or has applied for patents relating to the content of the manuscript?

- Do you have any other financial competing interests?
- Do you have any non-financial competing interests in relation to this paper?

If you can answer no to all of the above, write 'I declare that I have no competing interests' below. If your reply is yes to any, please give details below.

I declare that I have no competing interests

I agree to the open peer review policy of the journal. I understand that my name will be included on my report to the authors and, if the manuscript is accepted for publication, my named report including any attachments I upload will be posted on the website along with the authors' responses. I agree for my report to be made available under an Open Access Creative Commons CC-BY license (http://creativecommons.org/licenses/by/4.0/). I understand that any comments which I do not wish to be included in my named report can be included as confidential comments to the editors, which will not be published.

Choose an item.

To further support our reviewers, we have joined with Publons, where you can gain additional credit to further highlight your hard work (see: https://publons.com/journal/530/gigascience). On publication of this paper, your review will be automatically added to Publons, you can then choose whether or not to claim your Publons credit. I understand this statement.

Yes Choose an item.
